# Azologization of serotonin 5-HT_3_ receptor antagonists

**DOI:** 10.3762/bjoc.15.74

**Published:** 2019-03-25

**Authors:** Karin Rustler, Galyna Maleeva, Piotr Bregestovski, Burkhard König

**Affiliations:** 1 Institute of Organic Chemistry, University of Regensburg, 93053 Regensburg, Germany; 2 Aix-Marseille University, INSERM, INS, Institut de Neurosciences des Systèmes, 13005 Marseille, France; 3 Department of Normal Physiology, Kazan State Medical University, Kazan, Russia; 4 Institute of Neurosciences, Kazan State Medical University, Kazan, Russia

**Keywords:** azobenzene, 5-HT_3_R, ion currents, photopharmacology, serotonin

## Abstract

The serotonin 5-hydroxytryptamine 3 receptor (5-HT_3_R) plays a unique role within the seven classes of the serotonin receptor family, as it represents the only ionotropic receptor, while the other six members are G protein-coupled receptors (GPCRs). The 5-HT_3_ receptor is related to chemo-/radiotherapy provoked emesis and dysfunction leads to neurodevelopmental disorders and psychopathologies. Since the development of the first serotonin receptor antagonist in the early 1990s, the range of highly selective and potent drugs expanded based on various chemical structures. Nevertheless, on-off-targeting of a pharmacophore’s activity with high spatiotemporal resolution as provided by photopharmacology remains an unsolved challenge bearing additionally the opportunity for detailed receptor examination. In the presented work, we summarize the synthesis, photochromic properties and in vitro characterization of azobenzene-based photochromic derivatives of published 5-HT_3_R antagonists. Despite reported proof of principle of direct azologization, only one of the investigated derivatives showed antagonistic activity lacking isomer specificity.

## Introduction

5-Hydroxytryptamine (5-HT), commonly known as serotonin [1–2] or enteramine [3–4], is a monoamine neurotransmitter and hormone which is produced in the brain and in intestines and regulates a large variety of physiological functions in the mammalian central and peripheral nervous system [1,5]. In the central nervous system (CNS), it modulates sleep–wake cycles, emesis, appetite, mood, memory, breathing, cognition and numerous other functions [6–9]. In the gastrointestinal (GI) tract, it causes peristalsis via either smooth muscle contraction or enteric nerve depolarization [10]. It is also found in the platelets, where it is presumably involved in blood coagulation and vasoconstriction. Furthermore, serotonin is one of the first neurotransmitters to appear during development [11] and may have an organizing function in the development of the mammalian CNS being involved in cell division, differentiation, survival, neuronal migration [12–13] and synaptogenesis [14]. Dysfunction of the 5-HT receptor (5-HTR) signalling during early developmental stages my lead to altered cognitive ability, neurodevelopmental disorders, and increased incidence of psychopathologies such as autism and schizophrenia [15–16].

Serotonin operates via seven classes of 5-HT receptors of which six are G protein-coupled receptors (GPCRs) and only one, the 5-HT_3_R, is a ligand-gated cation channel [5–6,17]. When this receptor was identified and cloned [18–20], it became clear that 5-HT_3_ takes a unique position as pentameric ligand-gated cation-selective ion channel belonging to the Cys-loop receptor subfamily. In vertebrates, this family also includes nicotinic acetylcholine receptors (nAChRs), γ-aminobutyric acid type A receptors (GABA_A_Rs), and glycine receptors (GlyRs). To date, five subunits of the 5-HT_3_ receptor are identified (5-HT_3_A–5-HT_3_E) [21]. Functional receptors are either constructed as 5-HT_3_A homopentamers or as heteropentamers containing 5-HT_3_A and 5-HT_3_B receptor subunits [22–24].

5-HT_3_ receptors are highly expressed in the brainstem, especially in areas involved in the vomiting reflex and in the dorsal horn of the spinal cord [25]. These receptors are also expressed presynaptically providing regulation of the neurotransmitters release [21–22].

Besides targeting of 5-HT_3_Rs for the treatment of psychiatric disorders, they are object to counteract postoperative nausea and chemo-/radiotherapy provoked emesis [26–29]. In the early 1990s, the first potent and selective 5-HT_3_ receptor antagonist ondansetron was initially developed [26]. Since then the development of 5-HT_3_R antagonists progressed. The first-generation antagonists are structurally categorized in three major classes: (I) carbazole derivatives (e.g., ondansetron), (II) indazoles (e.g., granisetron), and (III) indoles (e.g., dolasetron) [26,30]. Generally, 5-HT_3_R antagonists share a basic amine, a rigid (hetero-)aromatic system and a carbonyl group or isosteric equivalent which is coplanar to the aromatic system. Although the antagonists show a general structural motive, they differ in their binding affinities, dose responses, and side effects [22].

To improve prospective antagonists and obtain a systematic tool for receptor investigation, spatial and temporal restriction of ligand binding and concomitant activity regulation is desirable. Fuelled by light, the growing field of photopharmacology provides a noninvasive method to trigger a drug’s pharmacological response on demand [31–33]. To introduce photoresponsiveness into a biological system, different approaches are feasible, e.g., the use of caged ligands (CL) [34–37], photoswitchable tethered ligands (PTLs) [38–40], photoswitchable orthogonal remotely tethered ligands (PORTLs) [41] or photochromic ligands (PCLs) [31,42]. The latter ones represent small molecules, which can either be engineered via extension of the chemical structure of a known pharmacophore towards a photochromic moiety or via replacement of certain parts of the biomolecule to generate a photochromic hybrid biomolecule. In this context, various photochromic scaffolds including dithienylethenes, fulgi(mi)des, and azobenzenes are investigated [31,42]. The latter ones were already discovered in 1834 by E. Mitscherlich [43] but it took around another 100 years till G. S. Hartley [44] revealed their photo-induced *trans–cis* isomerization representing the time of birth of the azobenzene photoswitch. Benefiting of their accessible synthesis, large change in polarity and geometry upon switching, excellent photochromic properties and tuneability, azobenzenes are amongst the most widely used photochromic scaffolds [31,42,45–47]. Since their first use in a biological environment in the late 1960s for the photoregulation of the enzymatic activity of chymotrypsin [48], their applications in biology widely expanded towards receptor control [49–52] and fields as bacterial growth [53], vision restoration [53–55], the respiratory chain [56] and lipids [57–58]. Owing to the reported serotonin antagonists’ chemical structures, the use of azobenzene as photochromic scaffold in the presented work seemed axiomatic. Therefore, the primary design of our photochromic derivatives is based on the direct “azologization” [59] of reported non-photochromic antagonists [60–61] via replacement of the benzene-ring connecting amide bond and thioether, respectively, by an azo bridge.

## Results and Discussion

### Design and synthesis of azobenzene-based photochromic modulators

**Scheme 1:**
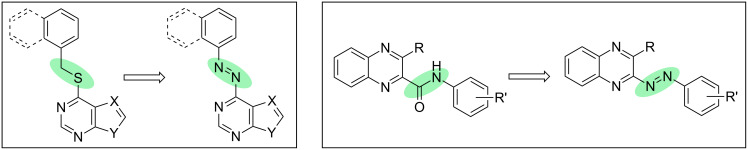
Approach of the direct azologization of reported [60–61] serotonin 5-HT_3_R antagonists via replacement of a thioether or amide bond by an azo bridge.

The reported [60–61] scaffolds of 5-HT_3_R antagonists are based on an aromatic system either connected to a purine/pyrimidine moiety via a thioether bridge or a quinoxaline moiety via an amide bond. Referring to this work performed by the groups of DiMauro [60] and Jensen [61], we envisioned that the replacement of the thioether or amide bond (Scheme 1) by an azo bridge would result in highly active photochromic serotonin 5-HTR antagonists controllable by irradiation with light. Based on the suggested receptor binding mode reported for one potent non-photochromic antagonist (lead structure of **16c**) [61] we expected the extended *trans*-isomer as biologically active configuration whereas its bent *cis*-isomer should be inactive.

### Synthesis of the quinoxaline-based azobenzenes

**Scheme 2:**
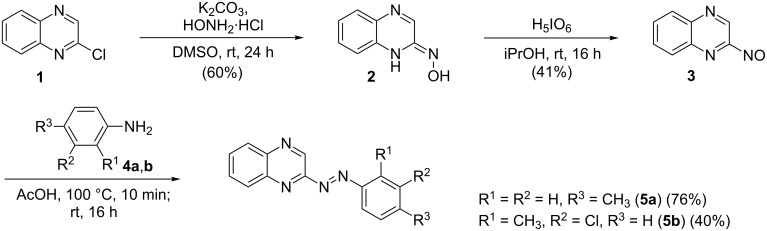
Synthesis of the differently substituted quinoxaline azobenzene derivatives **5a** and **5b** via Baeyer [62]–Mills [63] reaction [64–65].

The synthesis of the unsubstituted quinoxaline-based azobenzene derivatives **5a** and **5b** is based on a Baeyer [62]–Mills [63] reaction (Scheme 2). Therefore, nitrosoquinoxaline **3** was synthesized in a two-step procedure starting from 2-chloroquinoxaline (**1**), which was transformed into its oxime **2** using hydroxylammonium chloride [64]. The subsequent oxidation was performed using periodic acid as oxidant [65]. The subsequent reaction with differently substituted anilines in acetic acid [65] provided both quinoxaline azobenzene derivatives in good yields.

**Scheme 3:**
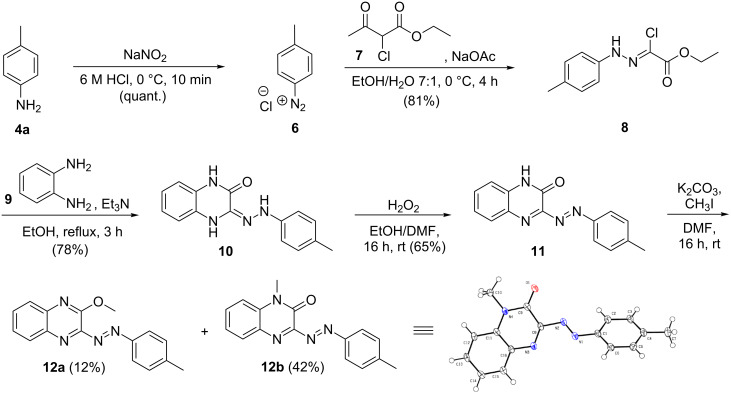
Synthesis of the methoxy-substituted quinoxaline derivative **12a** via diazotization [66–69].

The methoxy-substituted quinoxaline azobenzene derivative **12a** was synthesized via a different synthetic route depicted in Scheme 3. In a first step, *p*-toluidine (**4a**) was diazotized using sodium nitrite and subsequently reacted with the 2-chloroacetylacetone ester derivative **7** providing hydrazine **8** [66]. Upon reaction of the chloro-ester **8** with phenylenediamine (**9**) in the presence of triethylamine the quinoxaline moiety was formed [67]. Oxidation of the hydrazine derivative **10** using hydrogen peroxide under an oxygen atmosphere afforded the quinoxaline azobenzene derivative **11** [68]. Subsequent methylation using methyl iodide [69] mainly resulted in the formation of the *N*-methylated non-photochromic product **12b** but in low yields also the desired photochromic methoxy-substituted quinoxaline azobenzene derivative **12a**.

### Synthesis of the purine and thienopyrimidine-based derivatives

**Scheme 4:**
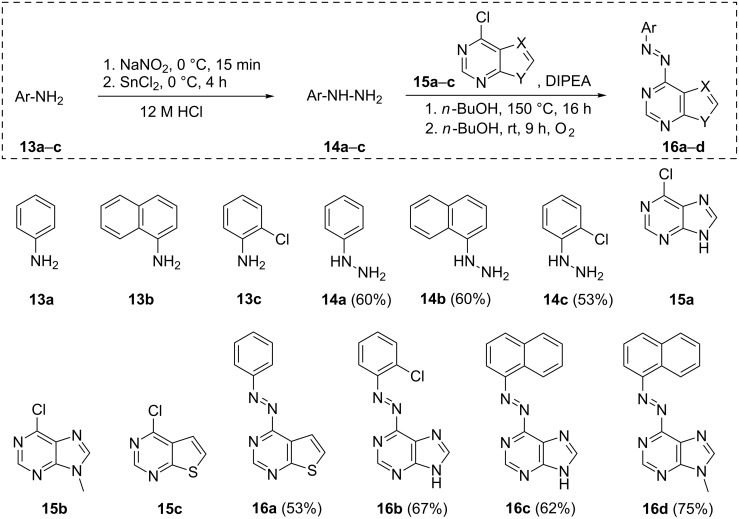
General procedure for the synthesis of purine- and thienopyrimidine-substituted arylazobenzenes and depiction of the corresponding structures [70–71].

Scheme 4 depicts the general procedure applied for the synthesis of differently substituted purine- and thienopyrimidine azobenzene derivatives. Differently substituted non-photochromic antagonists were chosen as lead structures delivering photochromic derivatives with varying electronic and thus photochromic properties. The respective arylamines **13a**–**c** were converted into their corresponding hydrazines **14a**–**c** via diazonium-salt formation using sodium nitrite and subsequent reduction using tin(II) chloride [70]. The following nucleophilic substitution at a chloro-substituted purine (**15a**,**b**) or thienopyrimidine (**15c**), respectively, and subsequent oxidation of the hydrazine moiety afforded the corresponding azobenzene derivatives **16a**–**d** [71].

### Synthesis of azobenzene-extended thiopurine derivatives

**Scheme 5:**
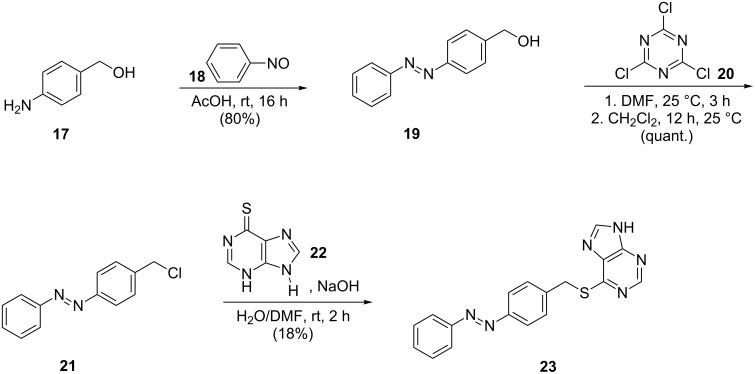
Synthesis of the thiomethyl-linked purine azobenzene **23** [62–63,72–74].

**Scheme 6:**
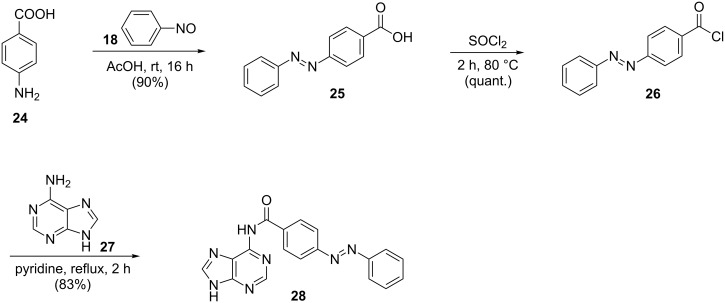
Synthesis of the amide-linked azobenzene purine **28** [62–63,75–77].

To further tune the photochromism and compare the properties of direct azologization to azo-extension, two additional derivatives of the in vitro most promising naphthalene azopurine **16c** were synthesized either by keeping the original thioether (Scheme 5) or replacing it by an amide bond (Scheme 6) known as common structural feature of 5-HT_3_R antagonists.

Scheme 5 reflects the synthesis of the azo-extended thiomethylpurine **23** starting with the synthesis of hydroxymethylazobenzene **19** [72] in a Baeyer [62]–Mills [63] reaction and subsequent nucleophilic substitution using cyanuric chloride (**20**) [73] providing chloromethyl azobenzene **21**. The introduction of the thiopurine moiety in **23** was accomplished upon reaction of **21** with dihydropurinethione **22** [74].

The amide-linked derivative of thiomethylpurine azobenzene **23** was synthesized via Baeyer [62]–Mills [63] formation of the carboxylated azobenzene **25** starting from aminobenzoic acid **24** and nitrosobenzene (**18**) [75]. Activation using thionyl chloride [76] afforded the acid chloride **26** and allowed amide-bond formation [77] for the generation of **28** (Scheme 6).

### Photochromic properties

**Figure 1:**
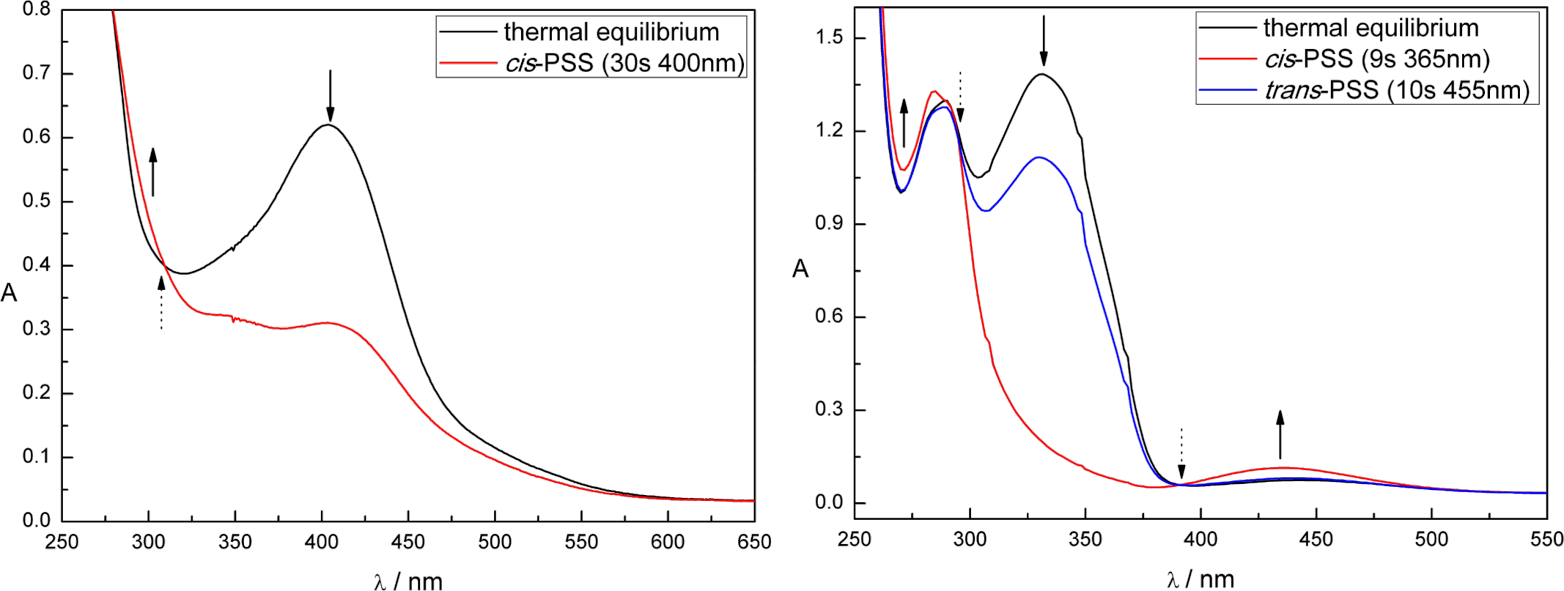
UV–vis absorption spectra measured at 50 µM in DMSO. Left: purine derivative **16c**; right: azo-extended derivative **23**.

The investigation of the photochromic properties of the potential 5-HT_3_R antagonists **5a**, **5b**, **12a**, **16a**–**d**, **23**, and **28** was performed in DMSO and depending on their solubility in phosphate buffer + 0.1% DMSO (**16a**–**d**) by UV–vis absorption spectroscopy. The compounds were dissolved at 50 µM in the respective solvent and irradiated with the indicated wavelengths to generate a substantial amount of their *cis*-isomer. This process can be followed by a decrease of the *trans*-absorption maximum at around 350–400 nm and an increase in absorption at around 450–500 nm in the UV–vis spectrum representing the *cis*-isomer (Figure 1, black arrows). The absorption bands of the *trans* and *cis*-isomers of compounds **12a**, **16c**, and **16d** overlap to such an extent, that no new maximum representing the *cis*-isomer was observed and thus *cis–trans* isomerization only occurs thermally and is not triggerable by irradiation with visible light. Back-isomerization was triggered by irradiation with visible light (**5a**, **5b**, **16a**, **16b**, **23**, and **28**) of the indicated wavelength or by thermal relaxation (**5a**, **5b**, **12a**, **16a**–**d**, **23**, and **28**). The irradiation times were determined by following the UV–vis spectrum upon isomerization until no more changes in absorption were observed and the photostationary state (PSS) was reached. The points of intersection in the absorption spectrum upon switching (= isosbestic points) indicate a clear two-component switching between *trans* and *cis*-species without any degradation or formation of a side-product (Figure 1, dotted black arrows). The UV–vis absorption spectra of all compounds are depicted in Supporting Information File 1, Figures S1–S10 and the data are summarized in Table S1 and Table S2. A comparison of the differently substituted purine azobenzene derivatives revealed the beneficial effect of an *o-*chloro substitution on the photochromic properties of **16b** compared to **16c** as the electron density at the nitrogen-rich purine core is reduced. Further reduction of the electron density was achieved by using a thienopyrimidine (**16a**) instead of a purine core (**16b–d**). Nevertheless, the photochromic properties of those heterocyclic, especially purine-based azobenzenes, are rather poor. In addition to direct azologization, two azo-extended purine derivatives **23** and **28** were synthesized resulting in excellent photochromic properties. Figure 1 compares exemplarily the UV–vis absorption spectra of the naphthalene-azo-purine **16c** (left) and its azo-extended azobenzene thioether purine **23** (right). The determination of the thermal half-lives (THL) of the *cis*-isomers of compounds **5a**, **5b**, **12a**, **16a–d**, **23**, and **28** was accomplished by monitoring the increase in absorbance which corresponds to the evolution of the *trans*-isomer after irradiation and exposure to dark. In contrast to the heterocyclic compounds **5a**, **5b**, **12a**, and **16a–d** with a thermal half-life in the seconds to minutes range, the azo-extended compounds **23** and **28** showed only slow thermal back-isomerization (day range) at room temperature. Depending on the desired application, both properties may be of benefit. For thermally instable compounds, only one wavelength for switching is required. In case of thermally stable *cis*-isomers constant irradiation to maintain a substantial amount of the *cis*-isomer can be avoided.

### Patch-clamp studies

**Figure 2:**
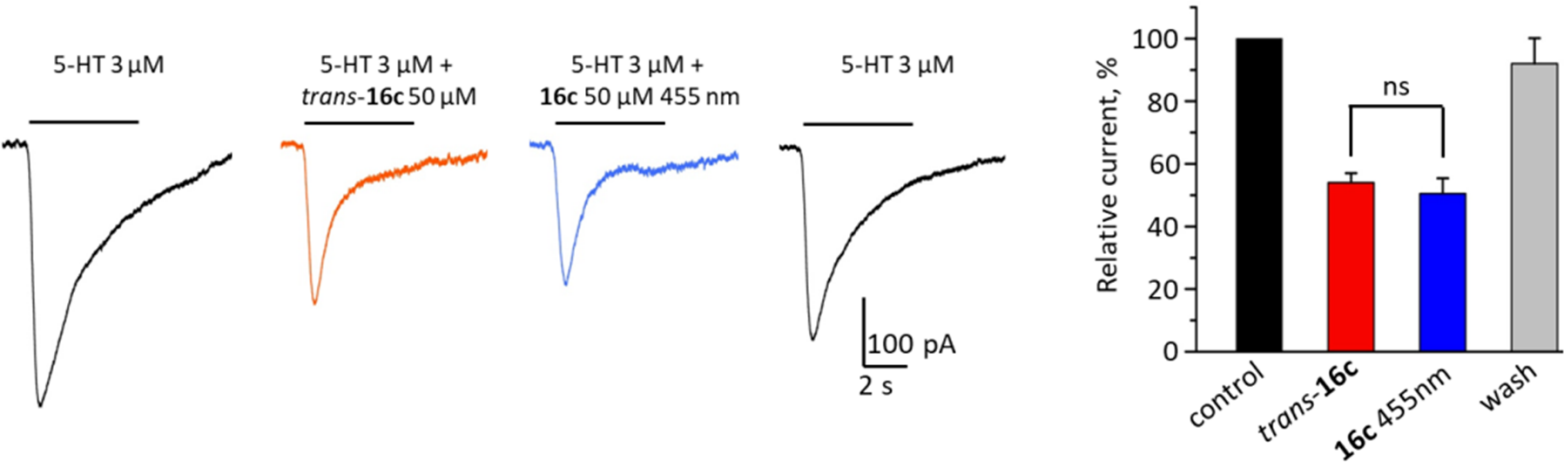
On the left panel representative traces of currents induced by the application of 3 µM 5HT (black trace), by 3 µM 5HT and 50 µM *trans*-**16c** (red trace), by 5HT and **16c** under constant irradiation (455 nm, blue trace), and again by pure 5HT – wash-out of the studied compound (black trace) are shown. On the right panel, a graph representing the relative amplitudes of currents in control (black column), at application of *trans*-**16c** (red column), at application of **16c** irradiated with blue light (blue column) and at wash-out (gray column) are shown. *P* > 0.05, paired t-test.

The synthesized azo antagonist derivatives **5a**, **5b**, **12a**, **16a–d**, **23**, and **28** were tested for their inhibitory activity using the patch-clamp technique on heterologously expressed ionotropic homopentameric 5-HT_3_A receptors. Only upon addition of **16c** the amplitude of the 5-HT_3_A mediated currents was decreased (Figure 2, left). Application of a 50 µM solution of *trans*-**16c** in its thermal equilibrium decreased the amplitude of 5-HT induced currents on 54 ± 3% (*n* = 4). However, irradiation-induced *trans–cis* isomerization with light of λ = 530 nm and 455 nm, respectively, had no significant effect on the amplitude of 5-HT_3_A-mediated currents (Figure 2, right).

## Conclusion

In the presented work, we address the design, synthesis, photochromic characterization and in vitro investigation of in total nine azobenzene-based derivatives of reported 5-HT_3_R antagonists. Initially, seven photoligands (**5a**, **5b**, **12a**, and **16a–d**) either based on quinoxaline (**5a**, **5b**, and **12a**) or purine derivatives (**16a–d**) with varying electronic and thus photochromic properties were synthesized by direct azologization of the respective leads. Especially the purine-based azobenzenes displayed high solubility in aqueous media. The beneficial effect of substituents reducing the overall electron density of the purine moiety (**16a**, **16b**) resulted in higher photostationary states and better band separation compared to **16c** and **16d**. Still, only one compound (**16c**) showed antagonistic activity in patch-clamp studies. This might be explained by the fact that its corresponding non-photochromic lead is the inhibitory most active reported [61] antagonist among the investigated ones. The partial rigidization of the thioether via incorporation of an azo bridge might result in a vast loss of activity. Thereby, azologization of the less potent leads resulted in complete loss of inhibitory activity (**5a**, **5b**, **12a**, **16a**, **16b**, **16d**) and only the originally most potent derivative **16c** kept recordable antagonistic activity. The missing significant difference in activity upon irradiation-induced *trans–cis* isomerization of **16c** is probably due to its moderate photochromic properties and slow *trans–cis* isomerization (Figure 1, left). During the patch-clamp analysis, the cells are continuously superfused with external solution resulting in a fast exchange of the surrounding media and co-applied tested compounds. Thus, the *cis*-PSS of **16c** might not be reached by irradiation within the short time of compound application despite continuous irradiation. Therefore, two azobenzene-extended derivatives (**23** and **28**) with improved photochromic properties were synthesized but lost antagonistic activity probably due to their increased steric demand.

In ongoing studies, detailed molecular modelling is used to design potential photochromic antagonists fitting the requirements of the receptor’s binding pocket. Regarding the analysis method, compounds will be optimized towards either thermally stable *cis*-isomers to be tested separately upon prior irradiation or faster switching compounds.

## Supporting Information

File 1 Detailed photochromic characterization (UV–vis absorption spectra, cycle performances, thermal half-lives) and NMR spectra of all synthesized compounds are provided. The file contains crystal structures of compounds **12b** and **16a** and experimental procedures.
