# Supplementary material for: Azologization of serotonin 5-HT3 receptor antagonists
Source: Beilstein J Org Chem. 2019 Mar 25;15:780–8. doi: 10.3762/bjoc.15.74 (PMC6444460; doi:10.3762/bjoc.15.74)
Supplement: File 1 — Detailed photochromic characterization (UV–vis absorption spectra, cycle performances, thermal half-lives) and NMR spectra of all synthesized compounds are provided. The file contains crystal structures of compounds 12b and 16a and experimental procedures. [file Beilstein_J_Org_Chem-15-780-s001.pdf]

BEILSTEIN JOURNAL OF ORGANIC CHEMISTRY

Supporting Information

for

Azologization of serotonin 5-HT_3 receptor antagonists

Karin Rustler, Galyna Maleeva, Piotr Bregestovski and Burkhard König

Beilstein J. Org. Chem. **2019,** *15,* 780–788. doi:10.3762/bjoc.15.74

Detailed photochromic characterization (UV–vis absorption spectra, cycle performances, thermal half-lives) and NMR spectra of all synthesized compounds are provided. The file contains crystal structures of compounds 12b and 16a and experimental procedures

License and Terms: This is a supporting information file under the terms of the Creative Commons Attribution License (http://creativecommons.org/licenses/by/4.0). Please note that the reuse, redistribution and reproduction in particular requires that the authors and source are credited.
The license is subject to the *Beilstein Journal of Organic Chemistry* terms and conditions: (https://www.beilstein-journals.org/bjoc)

Table of contents

UV–vis absorption spectra, cycle performances and thermal half-lives S2

Tabular summarized photochemical data S12

^{1}H and ^{13}C NMR spectra S13

X-ray structures S23

Experimental section S27

References S31

UV–vis absorption spectra, cycle performances and thermal half-lives.

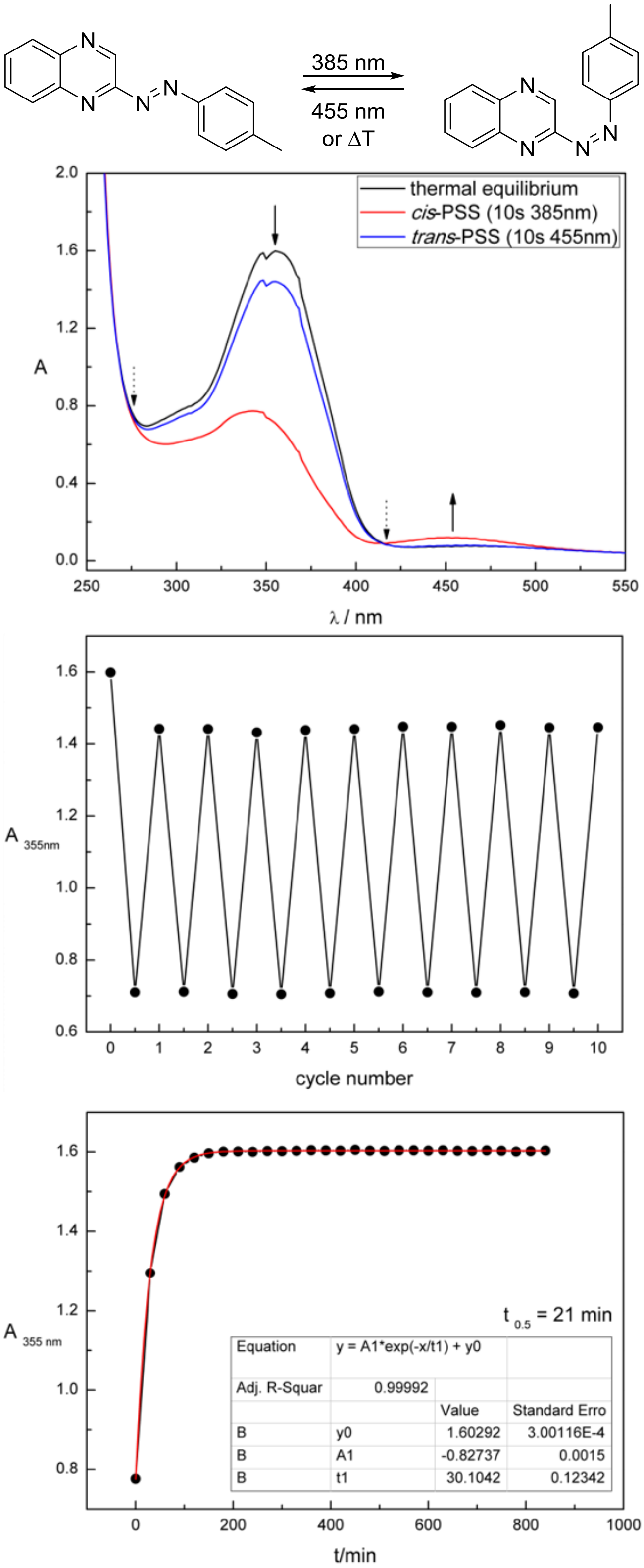

Figure S1: UV–vis absorption spectroscopic characterization of compound **5a** measured at 50 µM in DMSO. **Upper panel.** UV–vis absorption spectrum upon continuous irradiation with the indicated wavelengths until the PSS is reached. Black arrows indicate the changes in the absorption upon *trans*–*cis*-isomerization. Dotted black arrows indicate isosbestic points. **Middle panel.** Cycle performance. Changes in absorption at λ_{max} of the *trans*-isomer were measured during alternate

irradiation with the indicated wavelengths. **Lower panel.** Thermal half-life determined at λ_{max} of the *trans*-isomer.

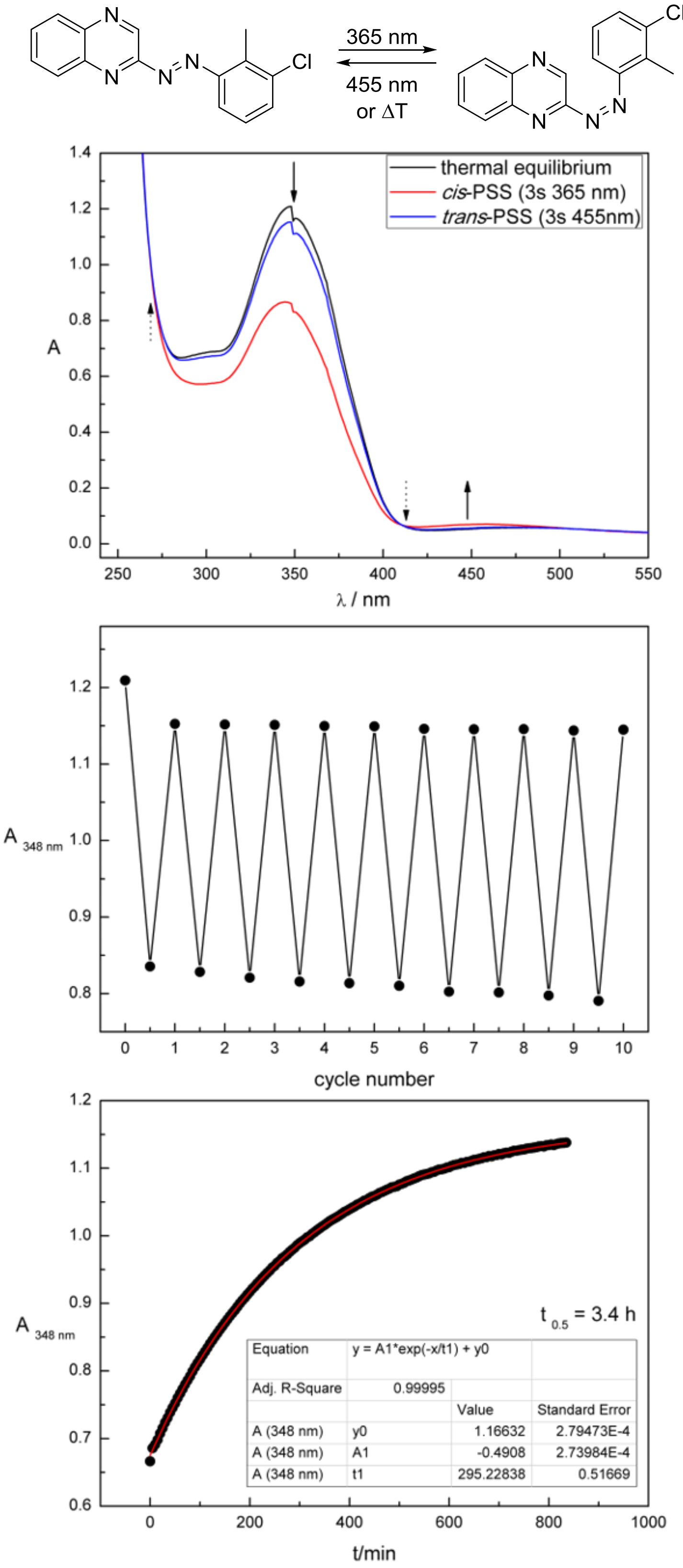

Figure S2: UV–vis absorption spectroscopic characterization of compound **5b** measured at 50 µM in DMSO. **Upper panel.** UV–vis absorption spectrum upon continuous irradiation with the indicated

wavelengths until the PSS is reached. Black arrows indicate the changes in the absorption upon *trans*–*cis*-isomerization. Dotted black arrows indicate isosbestic points. **Middle panel.** Cycle performance. Changes in absorption at λ_{max} of the *trans*-isomer were measured during alternate irradiation with the indicated wavelengths. **Lower panel.** Thermal half-life determined at λ_{max} of the *trans*-isomer.

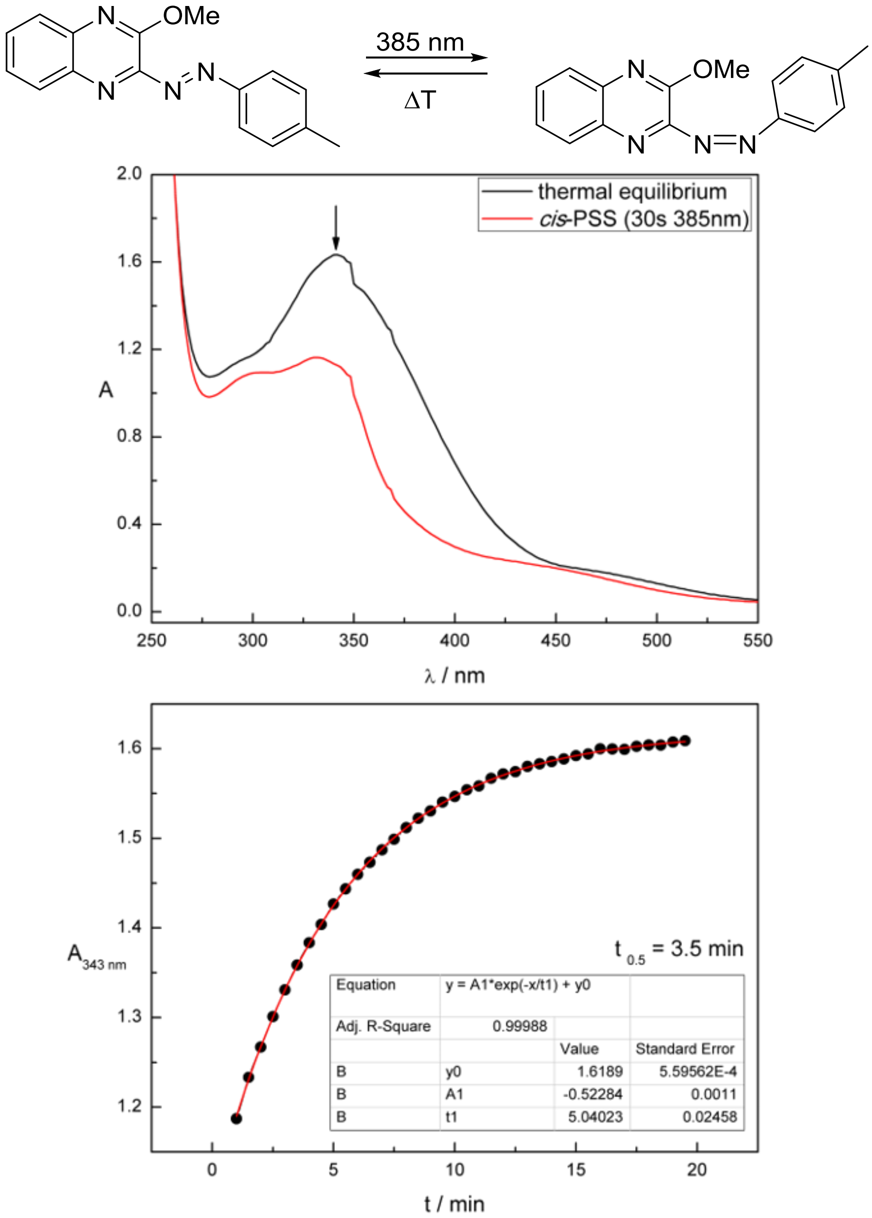

Figure S3: UV–vis absorption spectroscopic characterization of compound **12a** measured at 50 µM in DMSO. As the *cis*–*trans* back isomerization is not triggerable by irradiation with light no cycle performance was recorded. **Upper panel.** UV–vis absorption spectrum upon continuous irradiation with the indicated wavelength until the PSS is reached. Black arrow indicates the change in the absorption upon *trans*–*cis*-isomerization. **Lower panel.** Thermal half-life determined at λ_{max} of the *trans*-isomer.

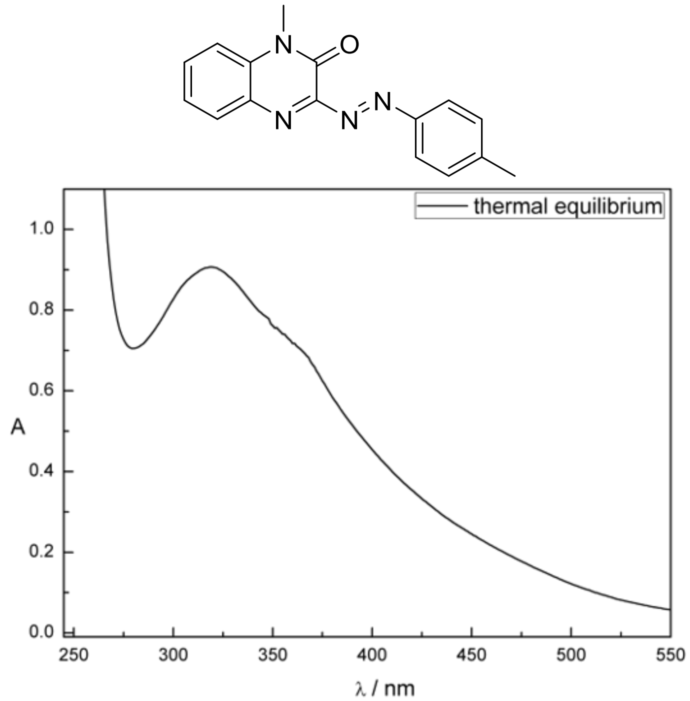

Figure S4: UV–vis absorption spectrum of non-photochromic compound **12b**.

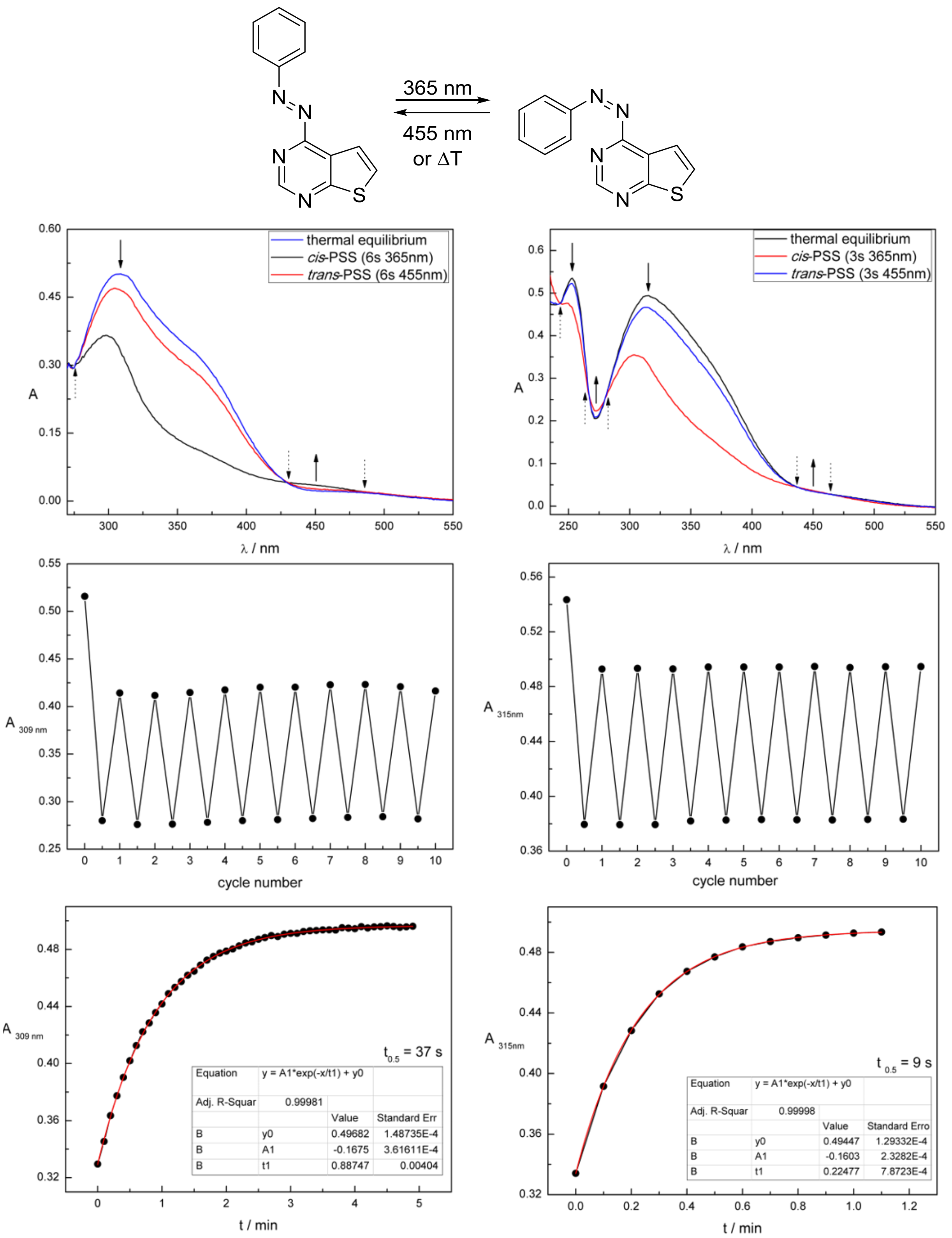

Figure S5: UV–vis absorption spectroscopic characterization of compound **16a** measured at 50 µM in DMSO (left) and phosphate buffer + 0.1% DMSO (right), respectively. **Upper panel.** UV–vis absorption spectra upon continuous irradiation with the indicated wavelengths until the PSS is reached. Black arrows indicate the changes in the absorption upon *trans–cis*-isomerization. Dotted black arrows indicate isosbestic points. **Middle panel.** Cycle performances. Changes in absorption at λ_{max} of the *trans*-isomer were measured during alternate irradiation with the indicated wavelengths. **Lower panel.** Thermal half-lives determined at λ_{max} of the *trans*-isomer.

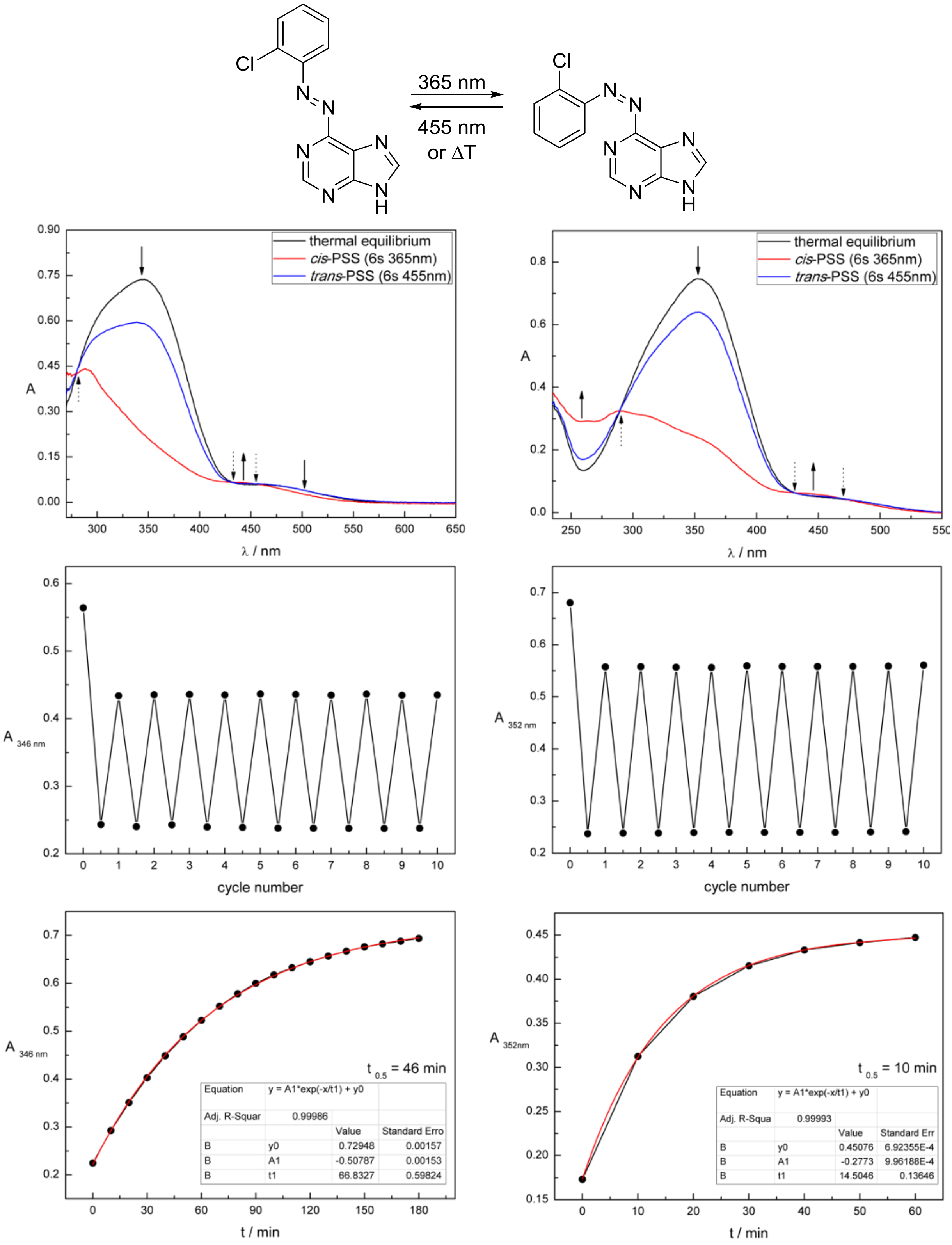

Figure S6: UV–vis absorption spectroscopic characterization of compound **16b** measured at 50 µM in DMSO (left) and phosphate buffer + 0.1% DMSO (right), respectively. **Upper panel.** UV–vis absorption spectra upon continuous irradiation with the indicated wavelengths until the PSS is reached. Black arrows indicate the changes in the absorption upon *trans*–*cis*-isomerization. Dotted black arrows indicate isosbestic points. **Middle panel.** Cycle performances. Changes in absorption at λ_{max} of the *trans*-isomer were measured during alternate irradiation with the indicated wavelengths. **Lower panel.** Thermal half-lives determined at λ_{max} of the *trans*-isomer.

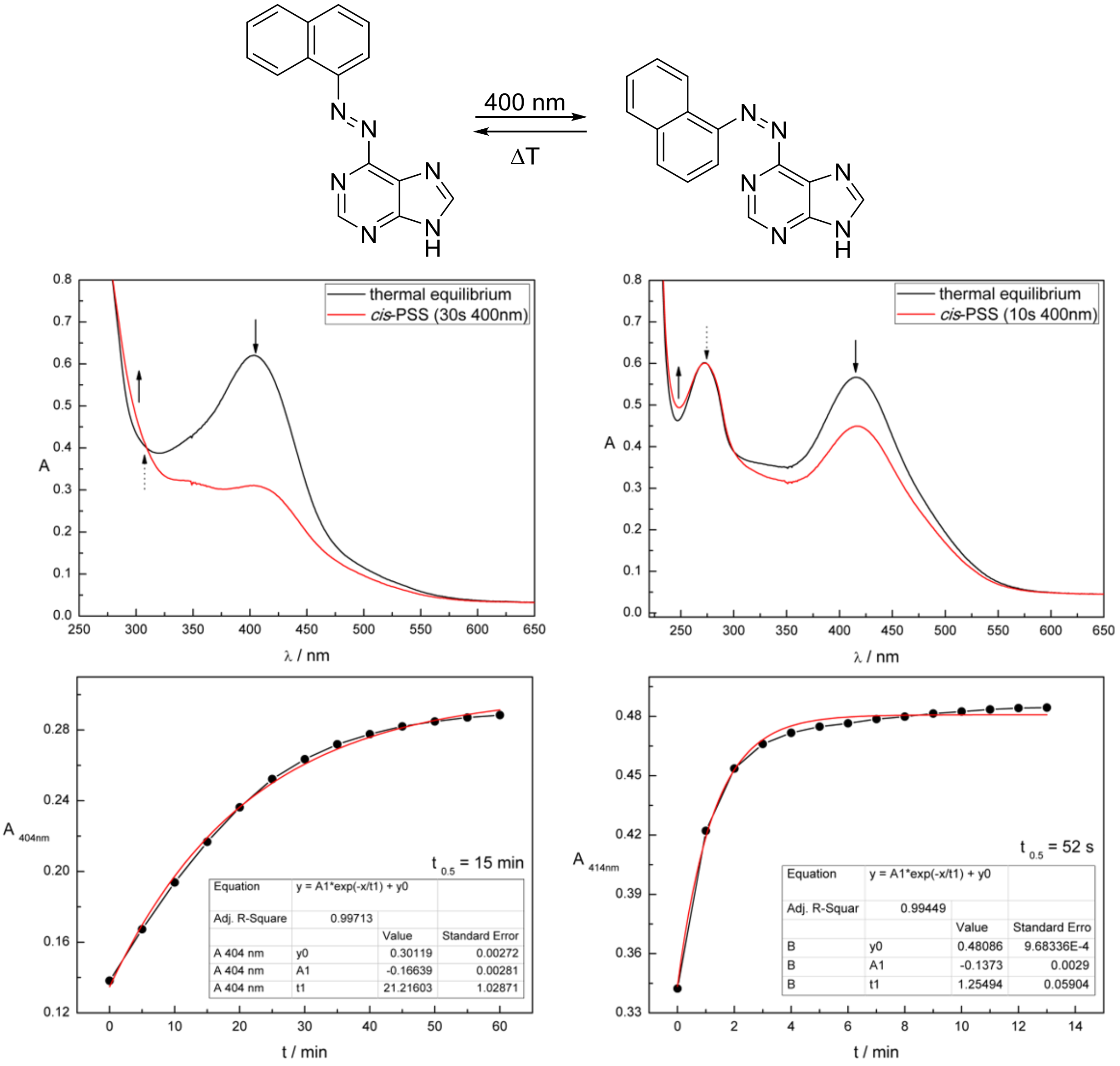

Figure S7: UV–vis absorption spectroscopic characterization of compound **16c** measured at 50 µM in DMSO (left) and phosphate buffer + 0.1% DMSO (right), respectively. As the *cis*–*trans* back isomerization is not triggerable by irradiation with light no cycle performances were recorded. **Upper panel.** UV–vis absorption spectra upon continuous irradiation with the indicated wavelength until the PSS is reached. Black arrows indicate the change in the absorption upon *trans*–*cis*-isomerization. **Lower panel.** Thermal half-lives determined at λ_{max} of the *trans*-isomer.

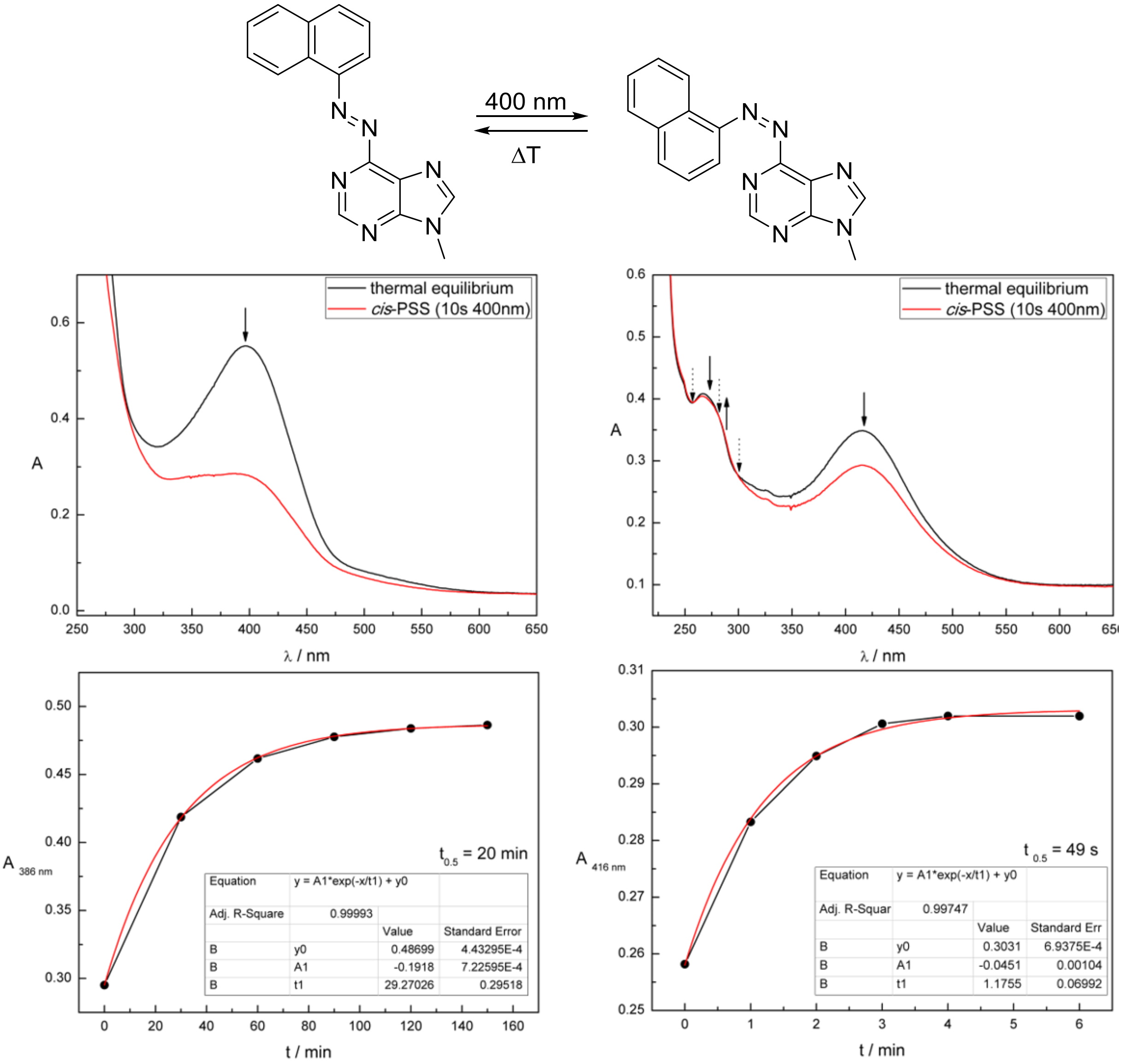

Figure S8: UV–vis absorption spectroscopic characterization of compound **16d** measured at 50 µM in DMSO (left) and phosphate buffer + 0.1% DMSO (right), respectively. As the *cis–trans* back isomerization is not triggerable by irradiation with light no cycle performances were recorded. **Upper panel.** UV–vis absorption spectra upon continuous irradiation with the indicated wavelength until the PSS is reached. Black arrows indicate the change in the absorption upon *trans–cis*-isomerization. Dotted black arrows indicate isosbestic points. **Lower panel.** Thermal half-lives determined at λ_{max} of the *trans*-isomer.

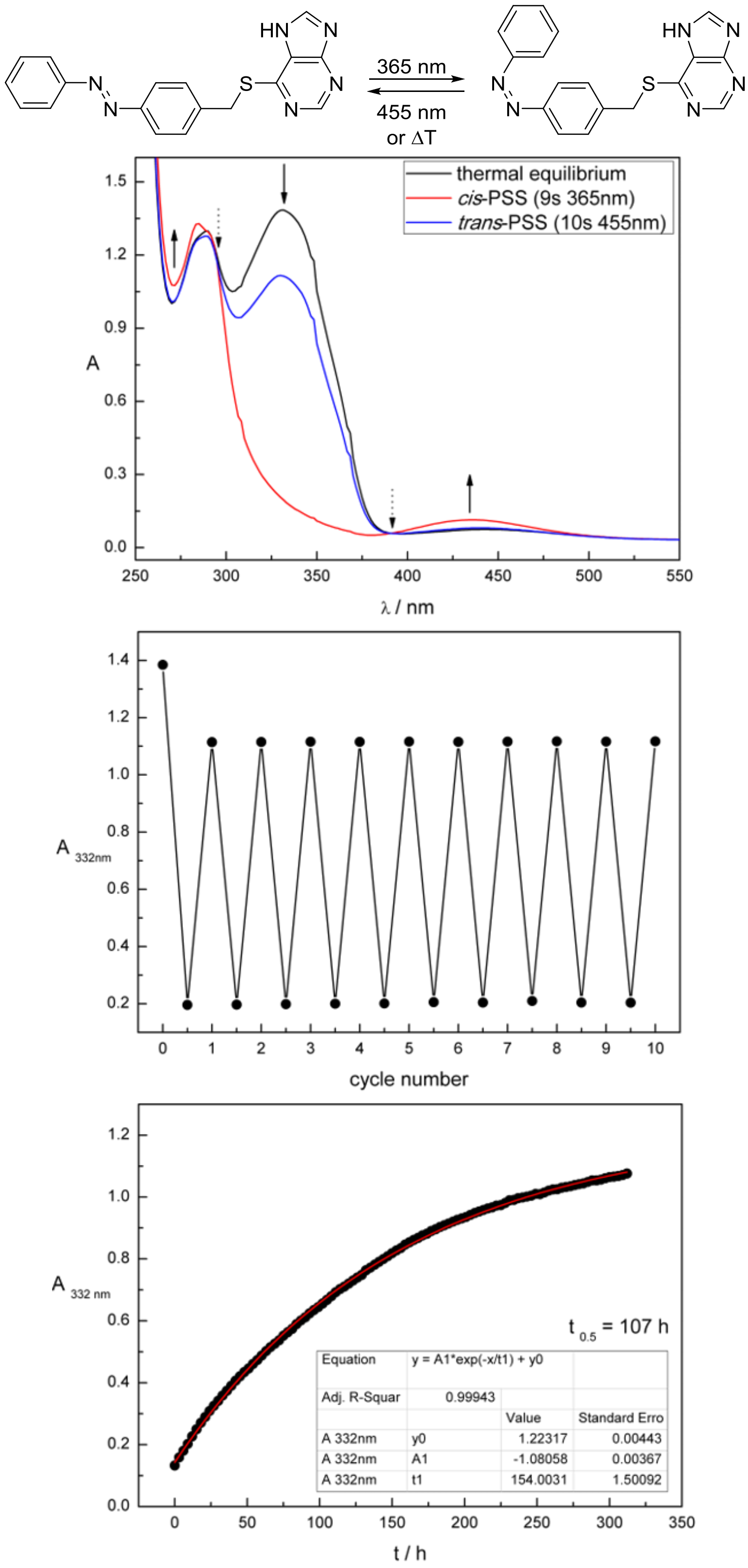

Figure S9: UV–vis absorption spectroscopic characterization of compound **23** measured at 50 µM in DMSO. **Upper panel.** UV–vis absorption spectrum upon continuous irradiation with the indicated wavelengths until the PSS is reached. Black arrows indicate the changes in the absorption upon *trans*–*cis*-isomerization. Dotted black arrows indicate isosbestic points. **Middle panel.** Cycle performance. Changes in absorption at λ_{max} of the *trans*-isomer were measured during alternate irradiation with the indicated wavelengths. **Lower panel.** Thermal half-life determined at λ_{max} of the *trans*-isomer.

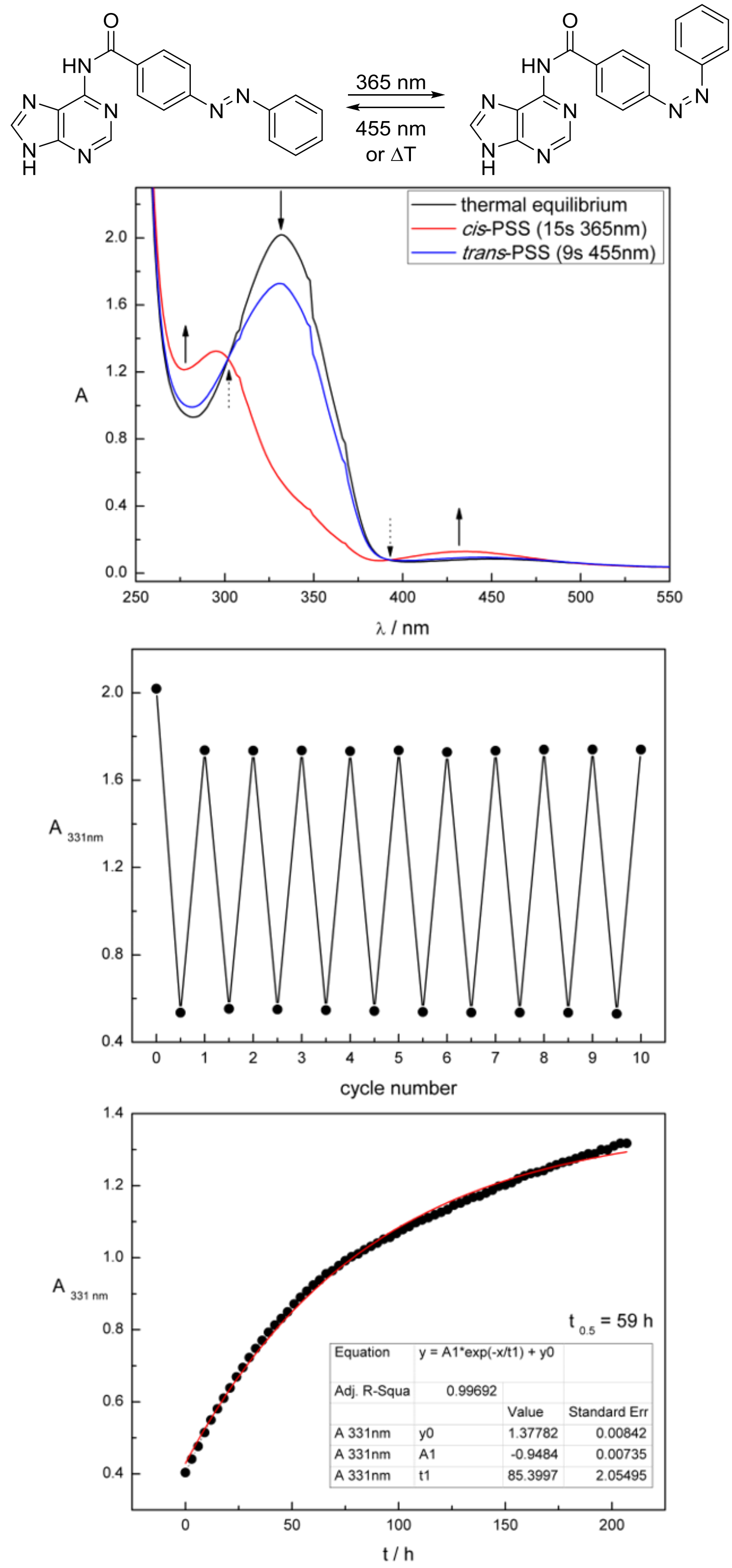

Figure S10: UV–vis absorption spectroscopic characterization of compound **28** measured at 50 µM in DMSO. **Upper panel.** UV–vis absorption spectrum upon continuous irradiation with the indicated wavelengths until the PSS is reached. Black arrows indicate the changes in the absorption upon *trans*–*cis*-isomerization. Dotted black arrows indicate isosbestic points. **Middle panel.** Cycle performance. Changes in absorption at λ_{max} of the *trans*-isomer were measured during alternate

irradiation with the indicated wavelengths. **Lower panel.** Thermal half-life determined at λ_{max} of the *trans*-isomer.

Tabular summarized photochemical data

Table S1: Photochemical properties of azobenzene-based serotonin 5-HT$_3$R antagonists determined at 50 µM in DMSO. Cpd = Compound. PSS-distribution determined by [a]NMR, [b]analytical HPLC measurement of a preirradiated sample at 20 °C.

Entry	Compound	λ_{max} *trans*-isomer [nm]	λ_{max} *cis*-isomer [nm]	isosbestic points [nm]	THL
1	**5a**	355	452	268, 415	21 min
2	**5b**	348	455	266, 411	3.4 h
3	**12a**	343	-	-	3.5 min
4	**12b**	319	-	-	-
5	**16a**	309	-	275, 430, 489	37 s
6	**16b**	346	-	279, 432, 459	46 min
7	**16c**	404	-	309	15 min
8	**16d**	386	-	309	20 min
9	**23**	332	437	289, 391	107 h
10	**28**	331	434	302, 392	59 h

Table S2: Photochemical properties of azobenzene-based serotonin 5-HT$_3$R antagonists determined at 50 µM in phosphate buffer + 0.1% DMSO.

Entry	Compound	λ_{max} *trans*-isomer [nm]	λ_{max} *cis*-isomer [nm]	isosbestic points [nm]	THL
1	**16a**	315	-	243, 267, 280, 438, 466	9 s
2	**16b**	352	-	289, 431, 470	10 min
3	**16c**	414	-	306	52 s
4	**16d**	416	-	314	49 s

Table S3: Determination of the photostationary states for the thermally more stable compounds **23** and **28** (at 50 µM in DMSO) using analytical HPLC at the isosbestic point at 20 °C.

Entry	Compound	PSS-distribution (50 µM in DMSO at 20 °C)
1	**23**	91% *cis* (365 nm); 81% *trans* (455 nm)
2	**28**	69% *cis* (365 nm); 84% *trans* (455 nm)

^{1}H and ^{13}C NMR spectra

Compound **5a** (DMSO-d_6)

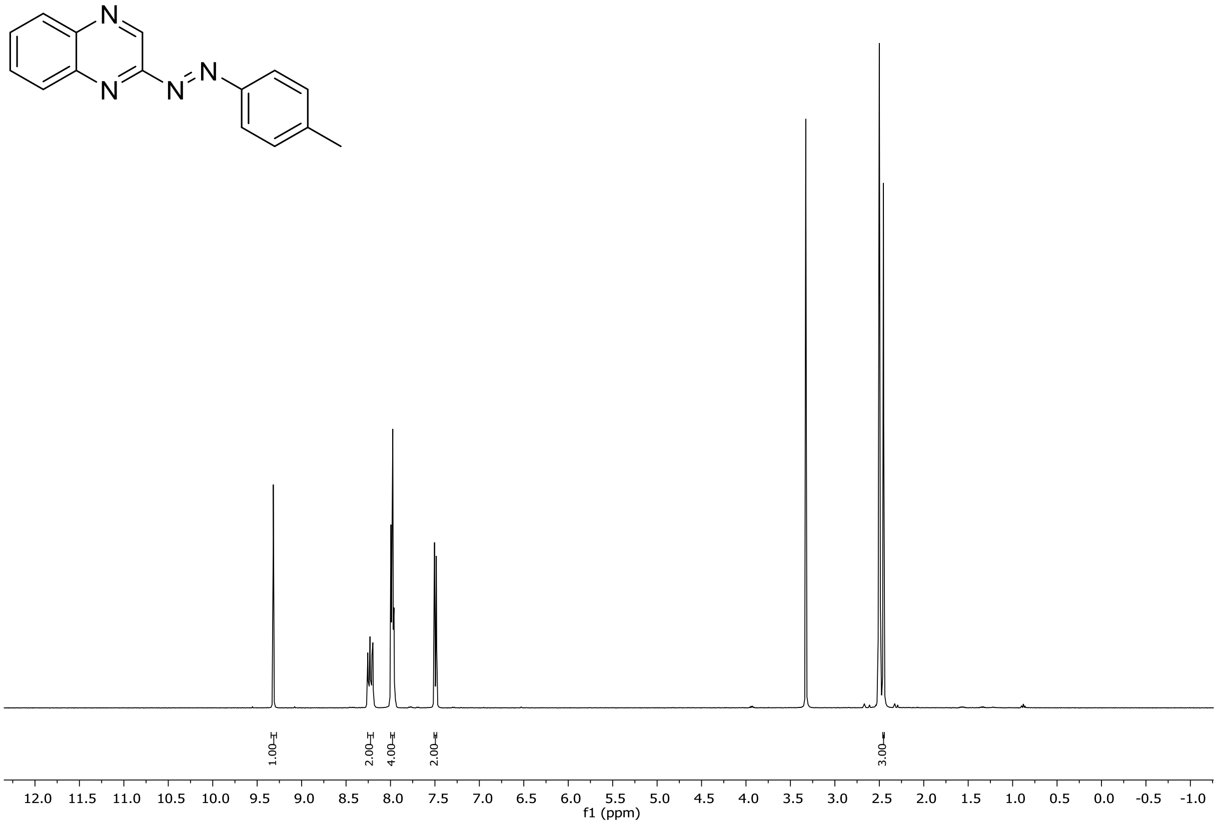

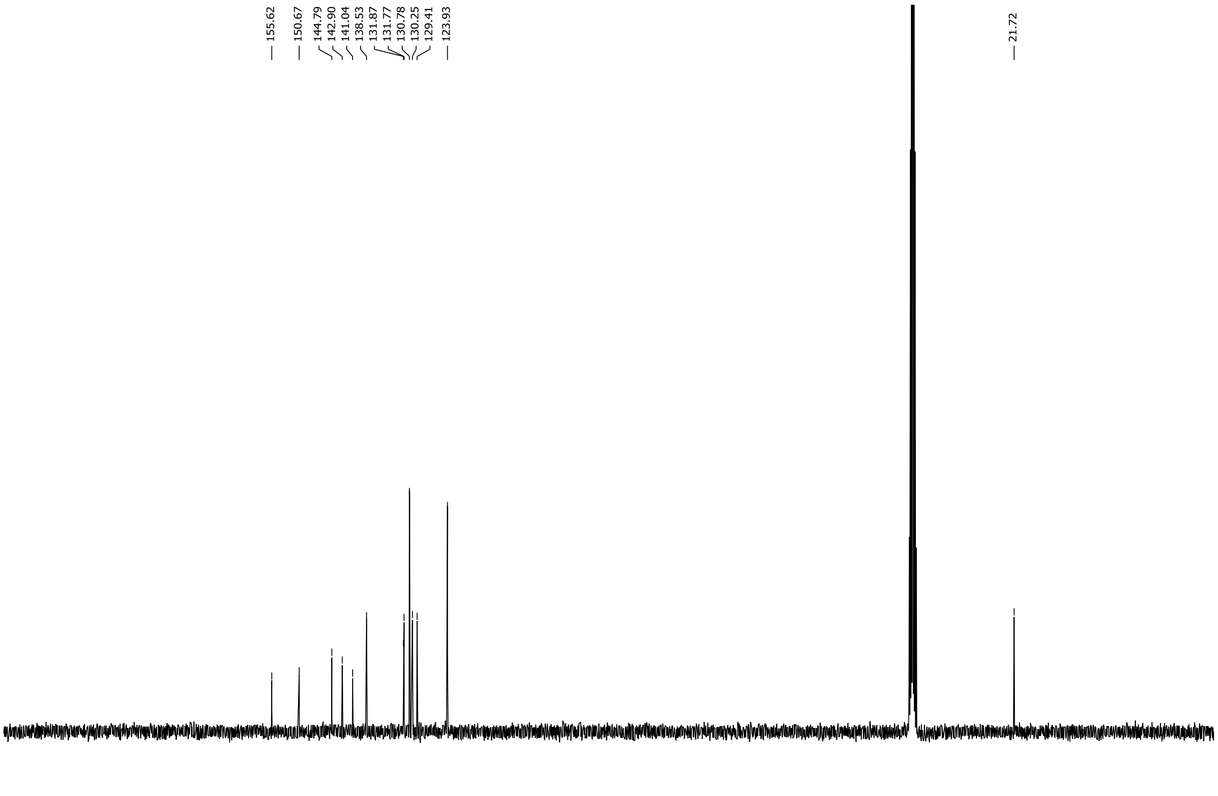

^{1}H and ^{13}C NMR spectra.

Compound **5b** (DMSO-d_6)

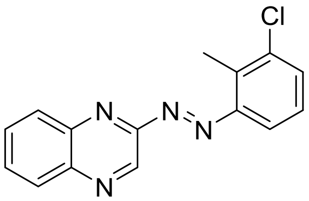

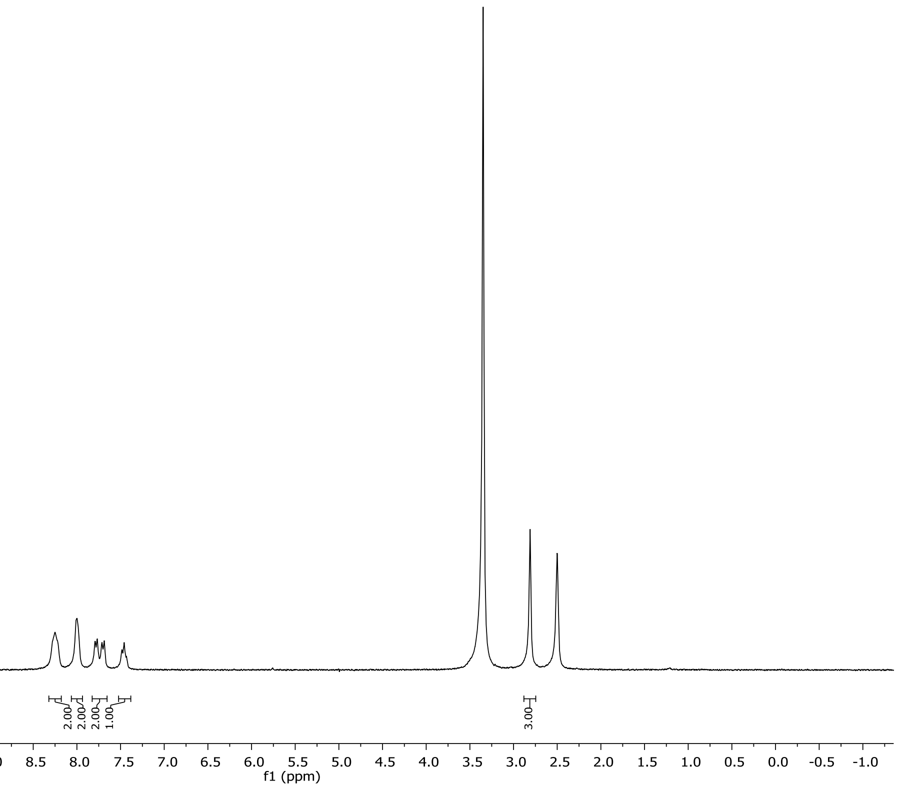

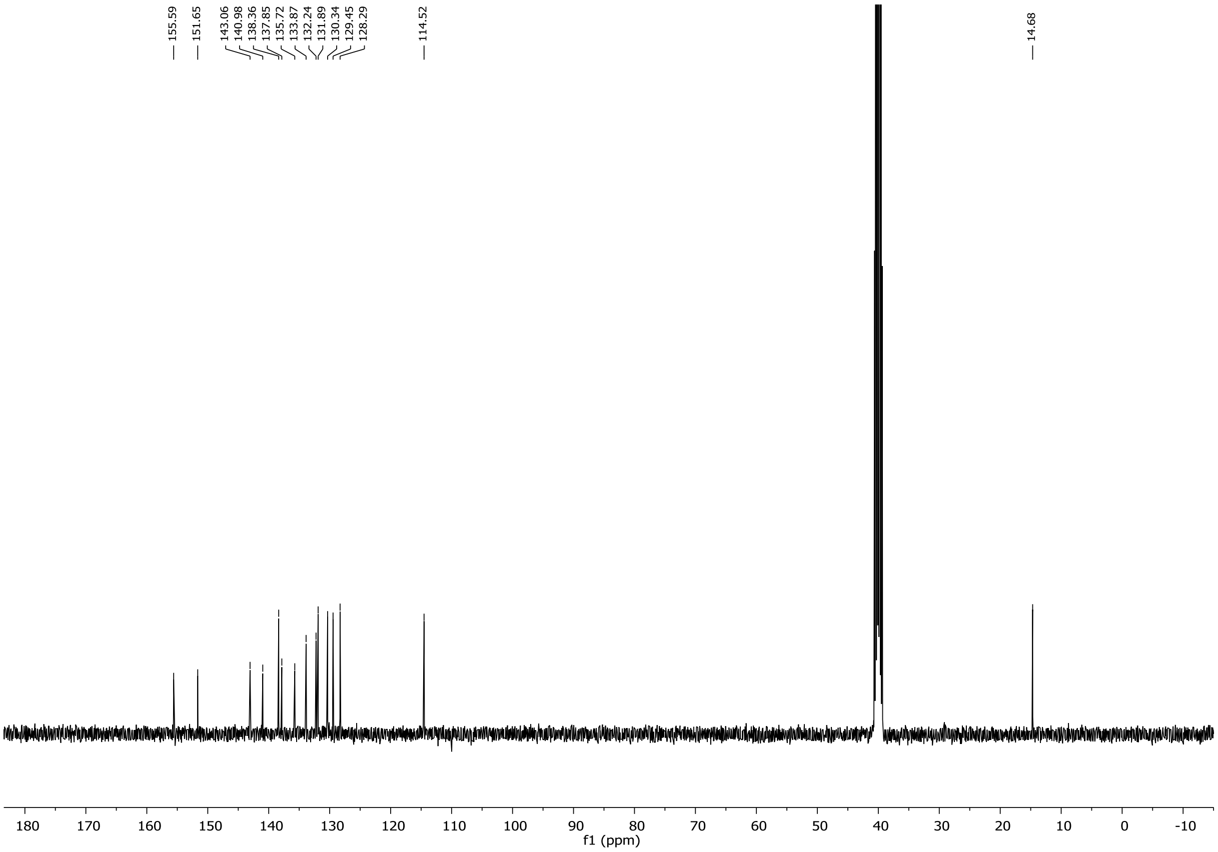

^{1}H and ^{13}C NMR spectra.

Compound **12a** (^{1}H DMSO-d_6, ^{13}C $CDCl_3$)

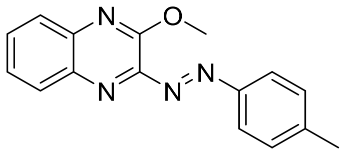

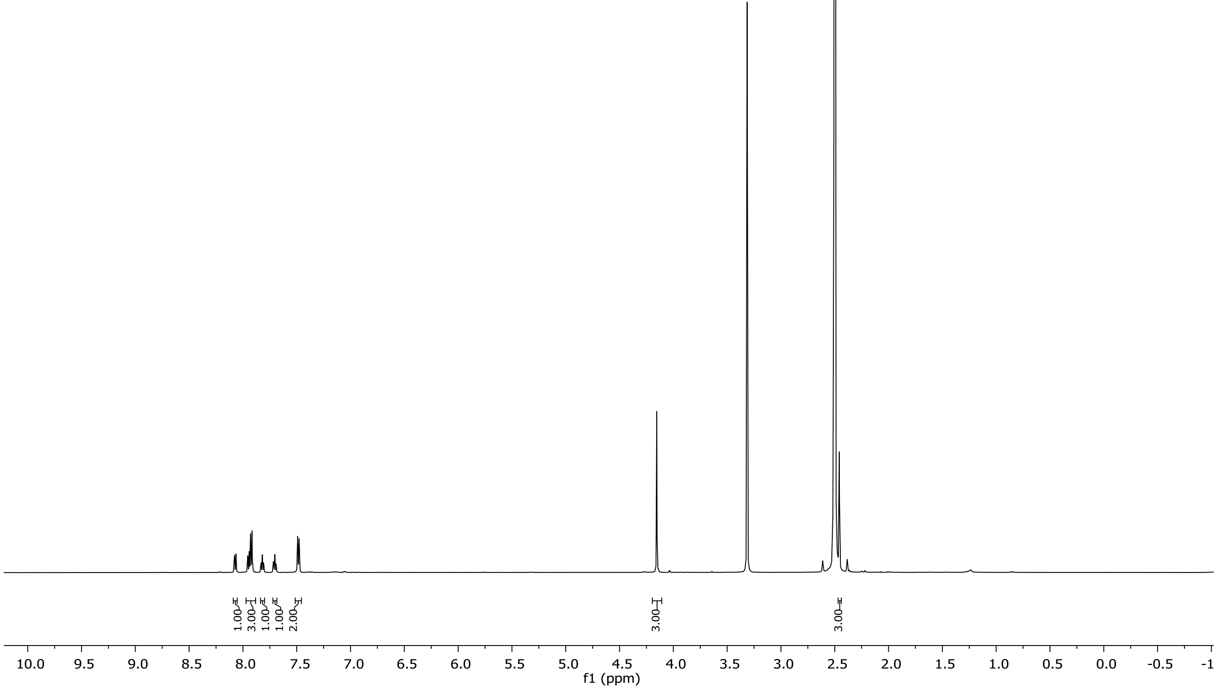

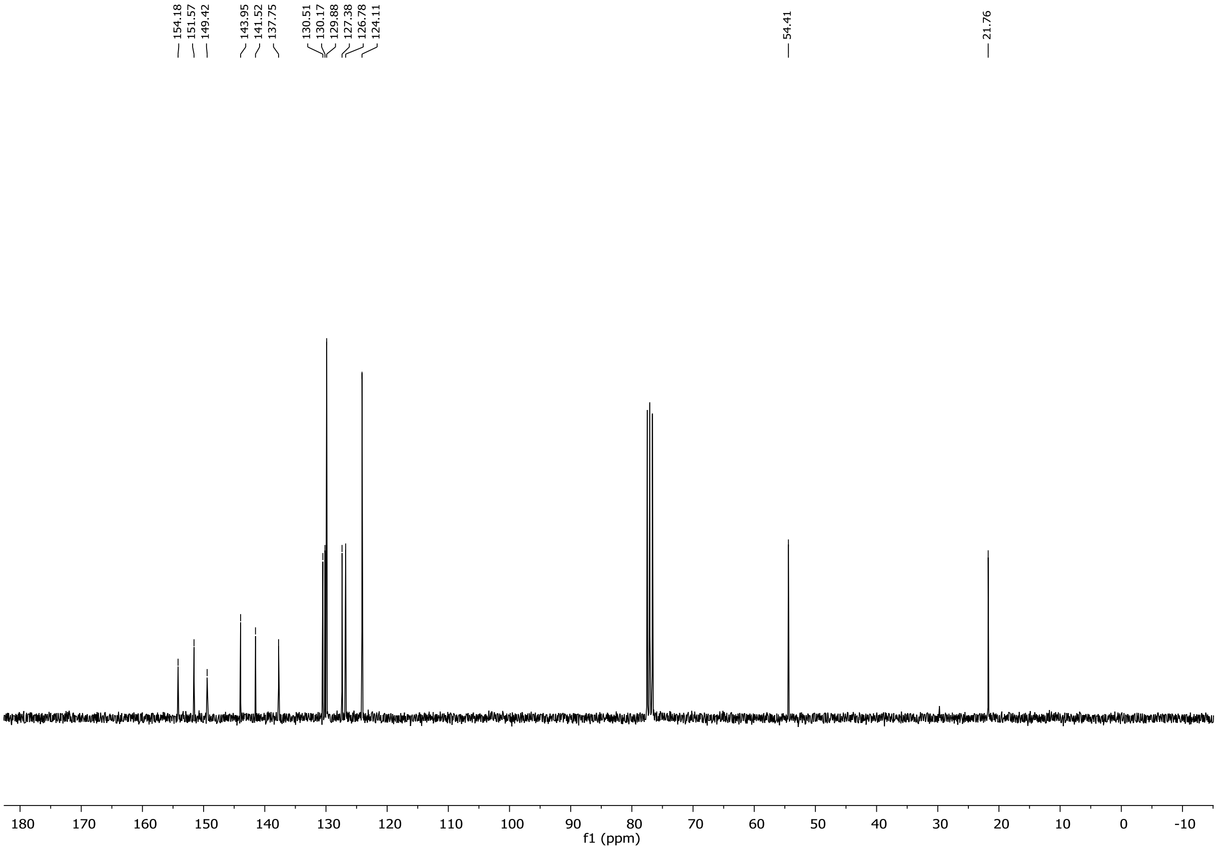

^{1}H and ^{13}C NMR spectra.

Compound **12b** (DMSO-d_6)

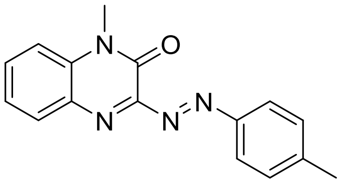

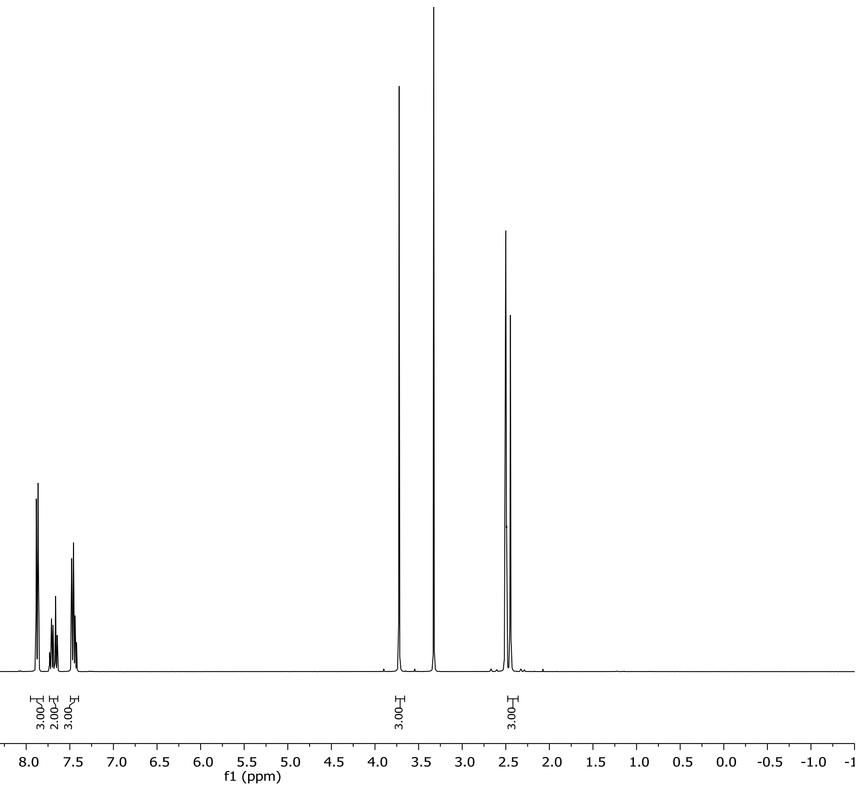

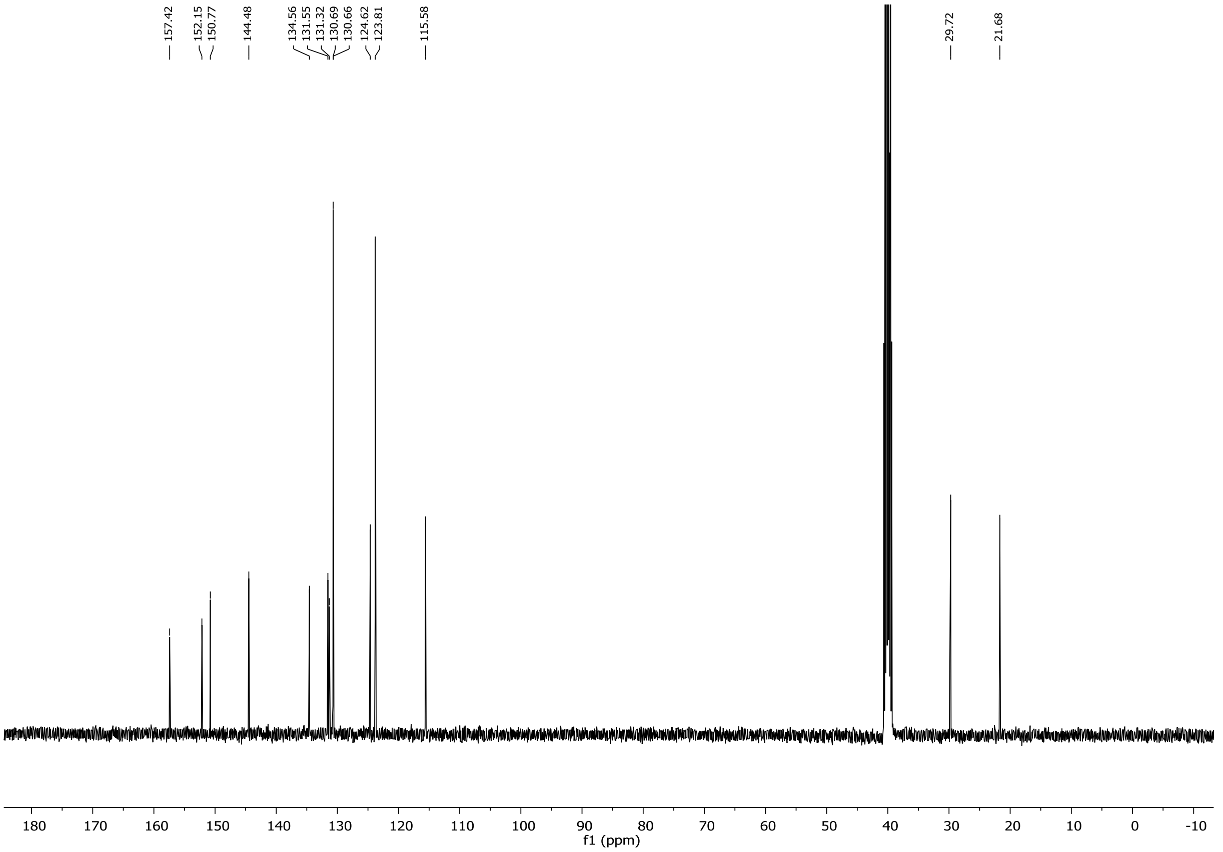

^{1}H and ^{13}C NMR spectra.

Compound **16a** (DMSO-d_6)

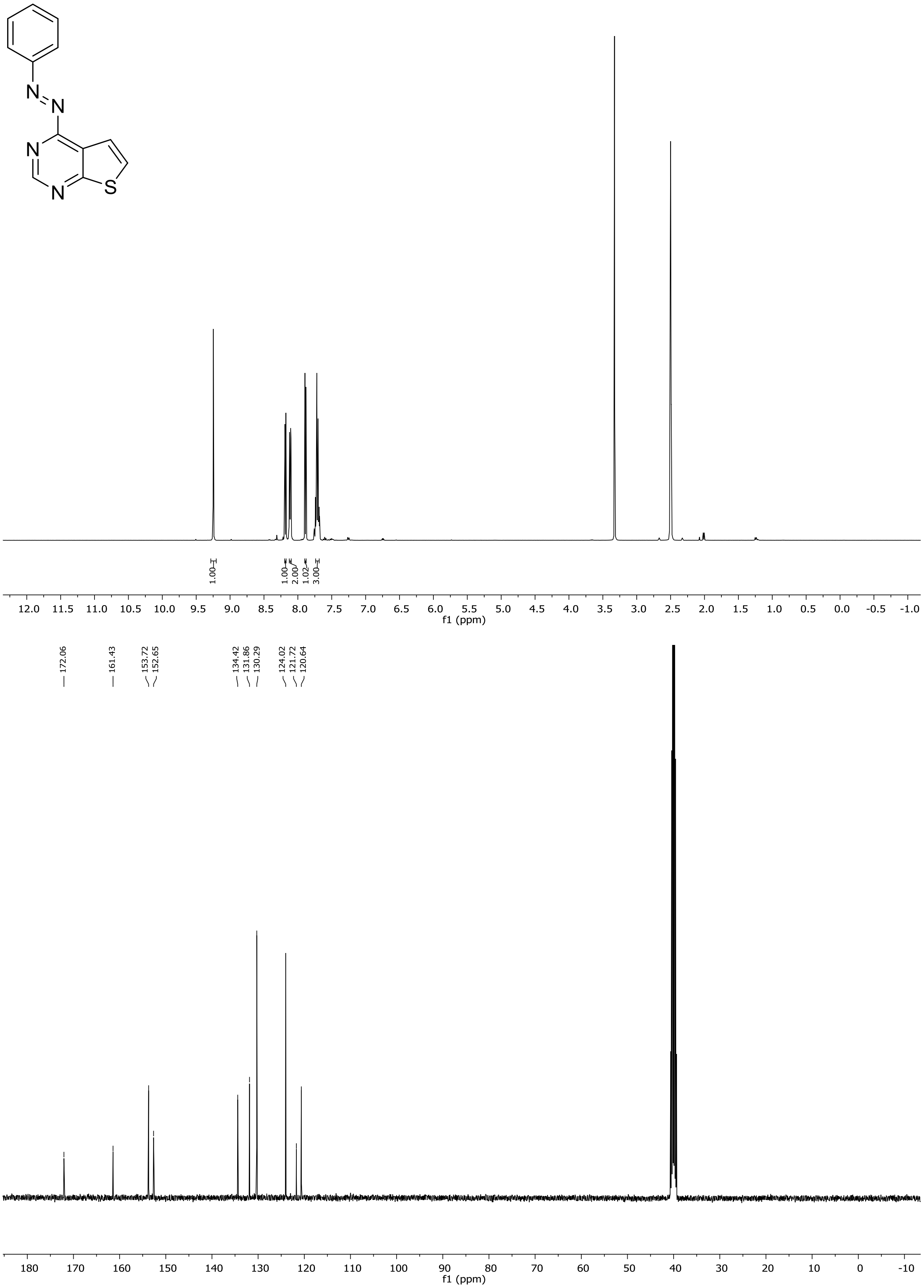

^{1}H and ^{13}C NMR spectra.

Compound **16b** (DMSO-d_6)

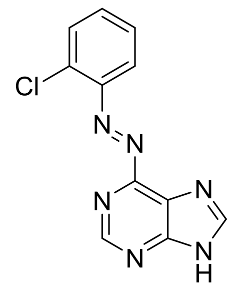

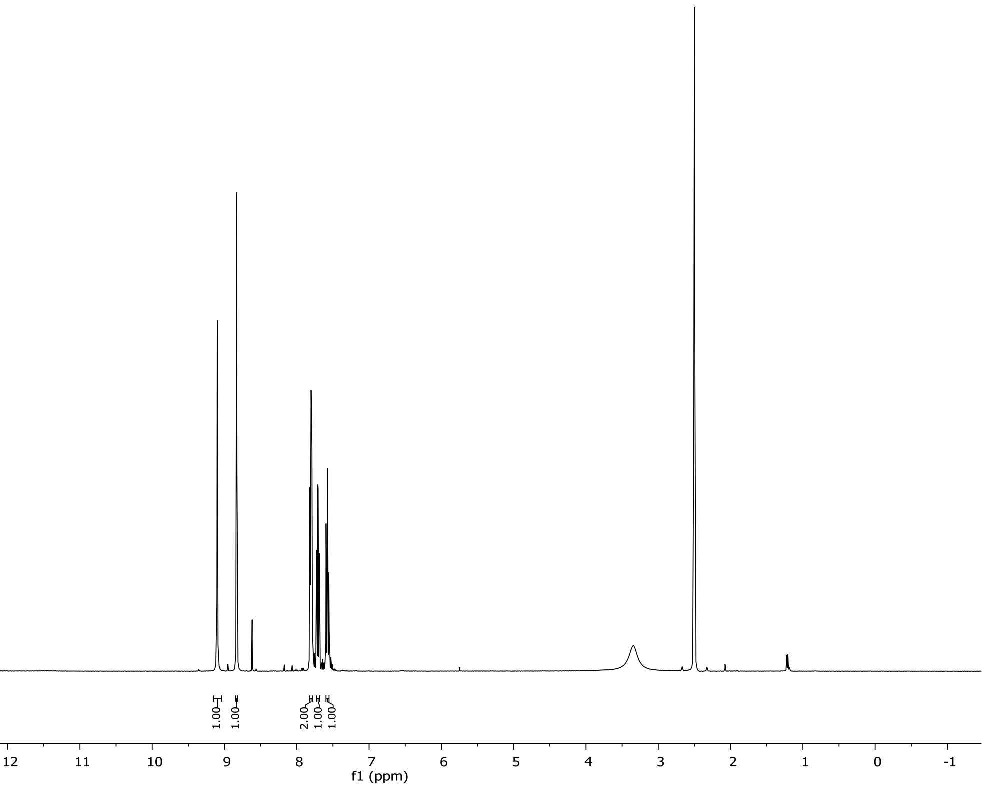

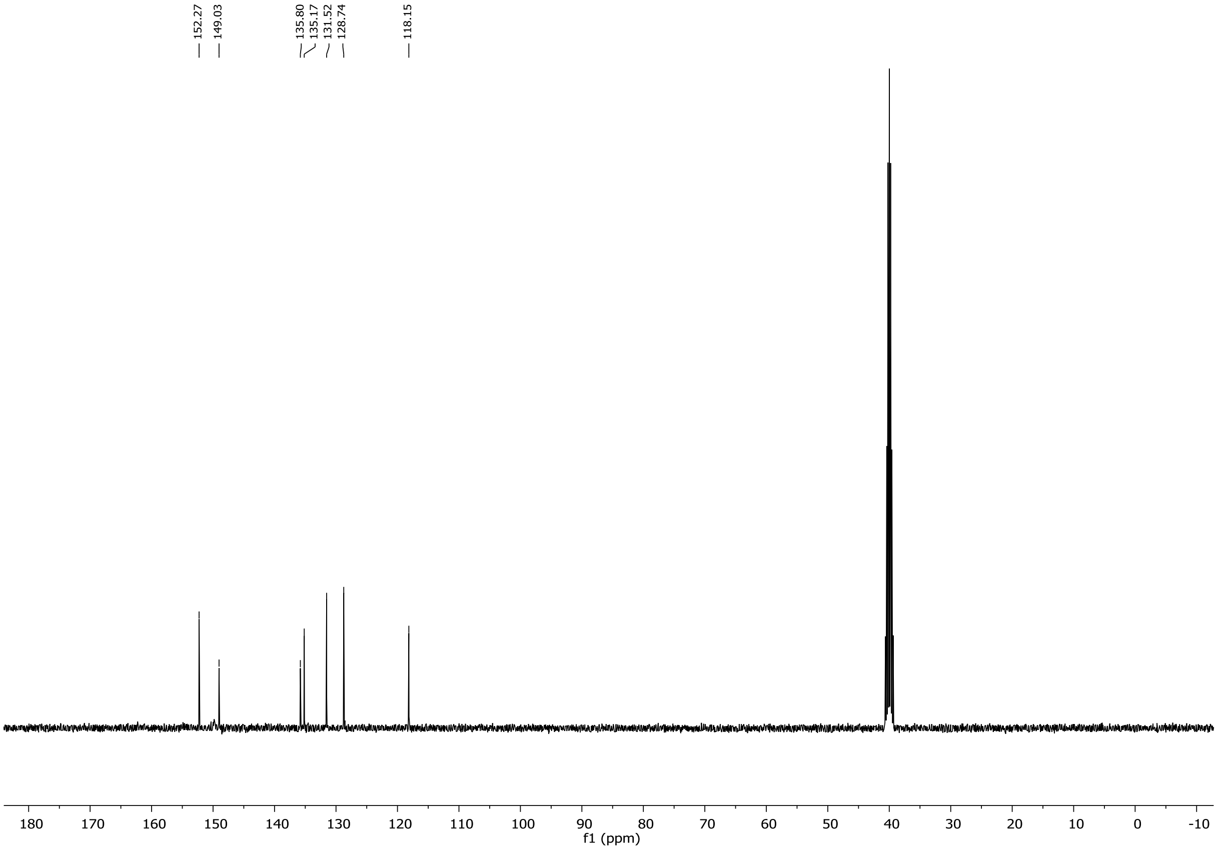

^{1}H and ^{13}C NMR spectra.

Compound **16c** (DMSO-d_6)

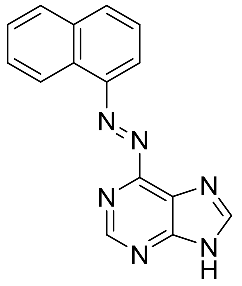

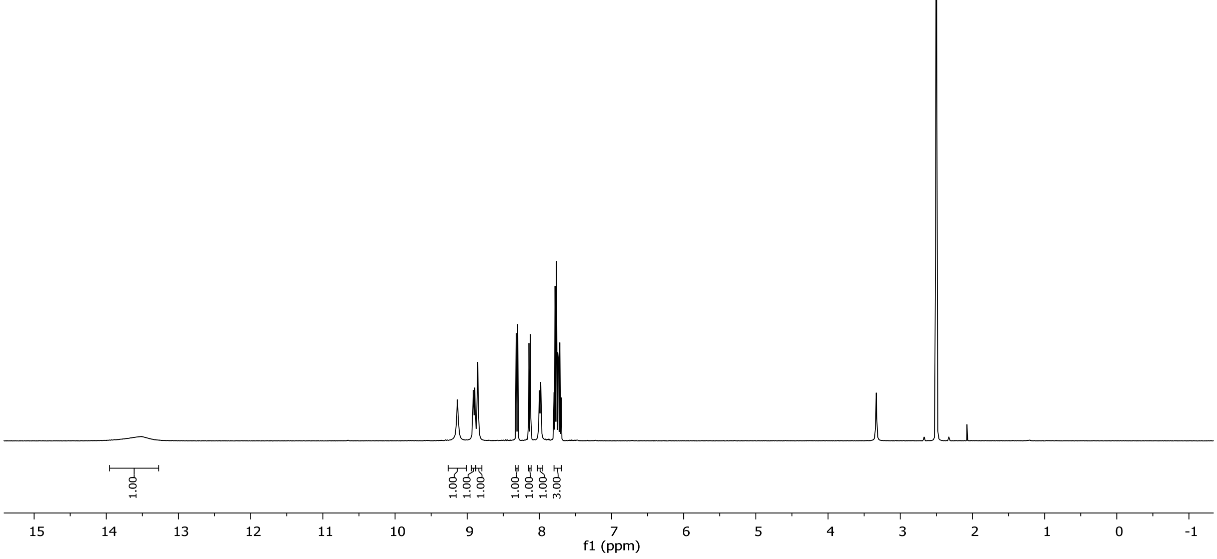

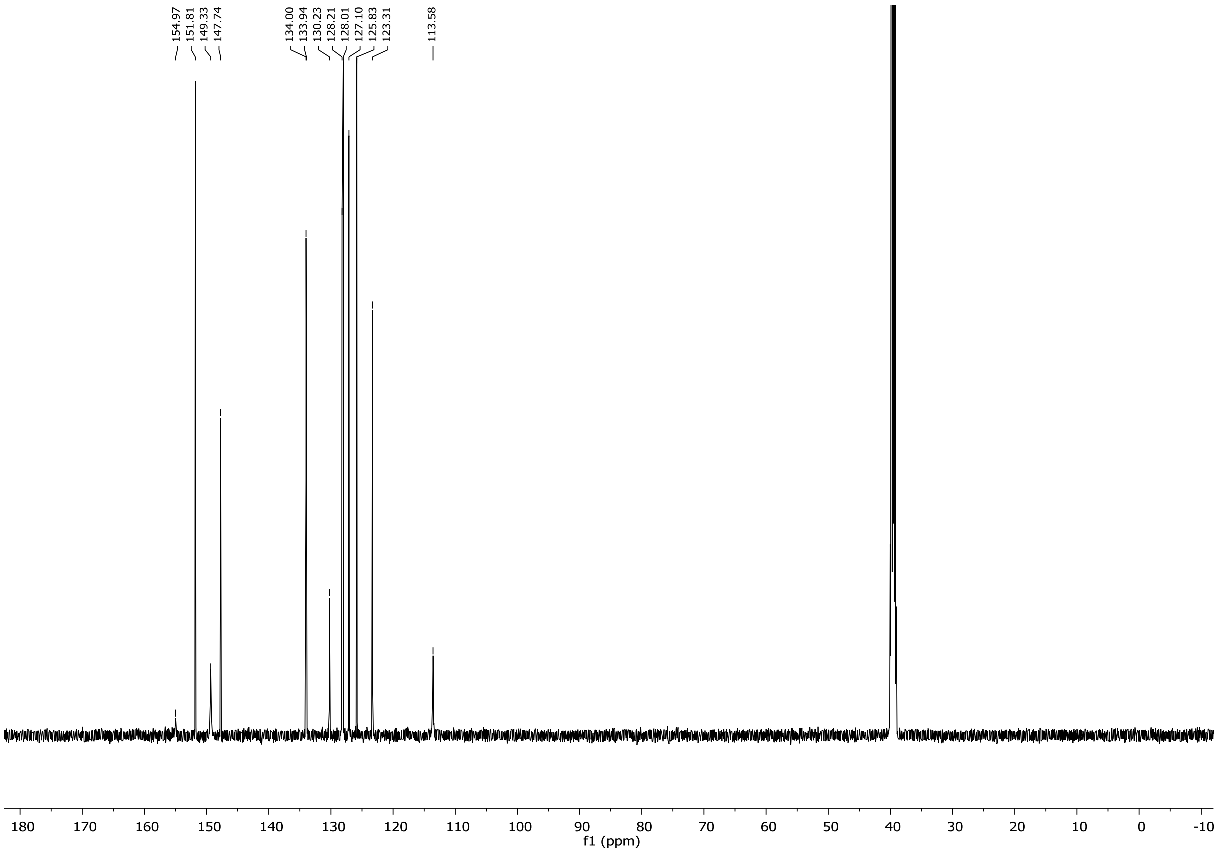

^{1}H and ^{13}C NMR spectra.

Compound **16d** ($CDCl_3$)

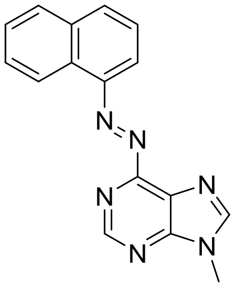

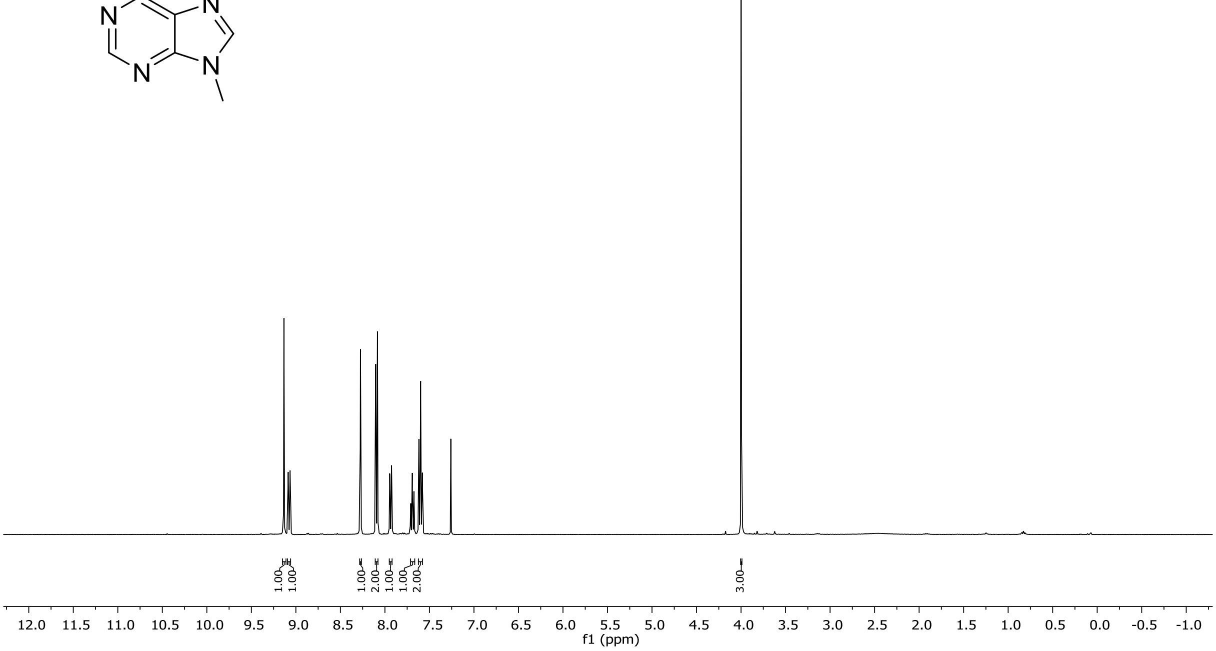

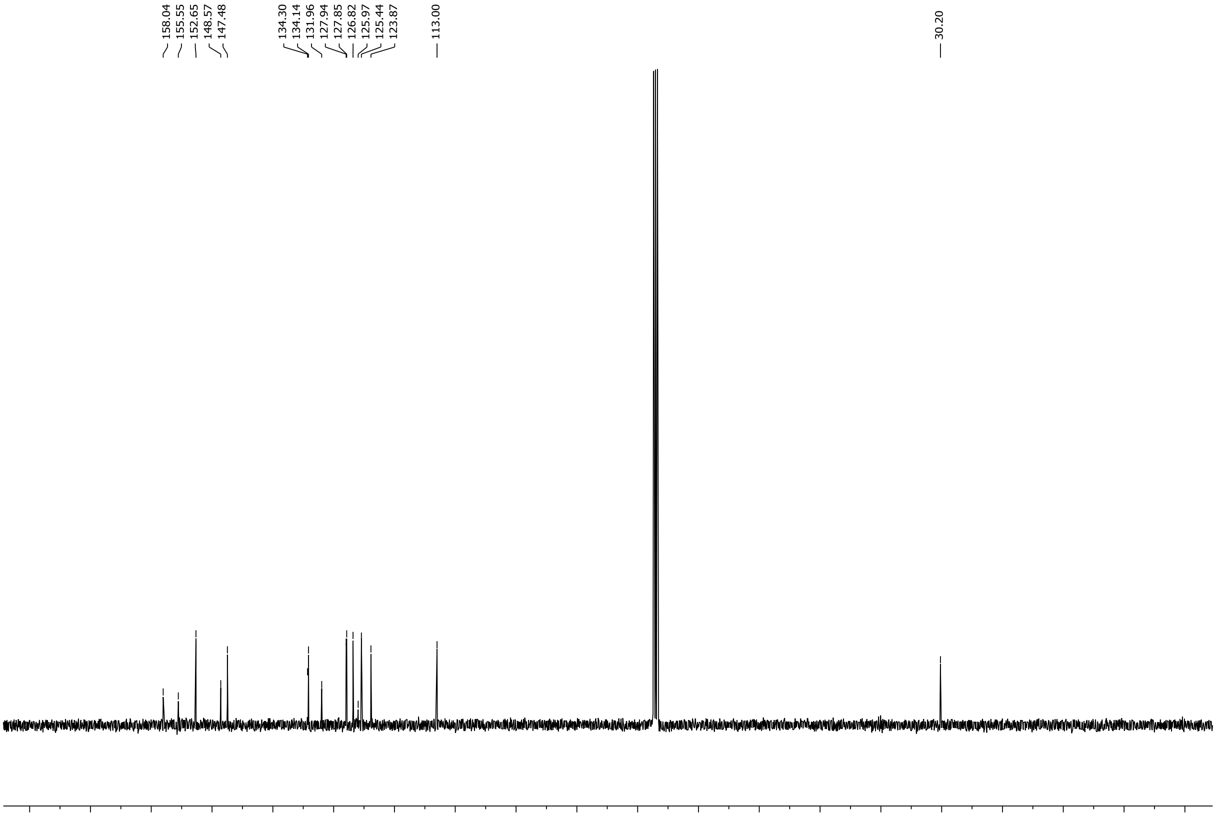

^{1}H and ^{13}C NMR spectra.

Compound **23** (DMSO-d_6)

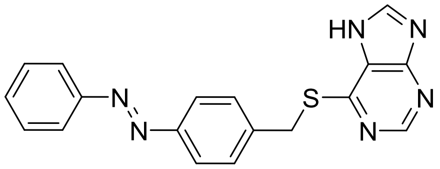

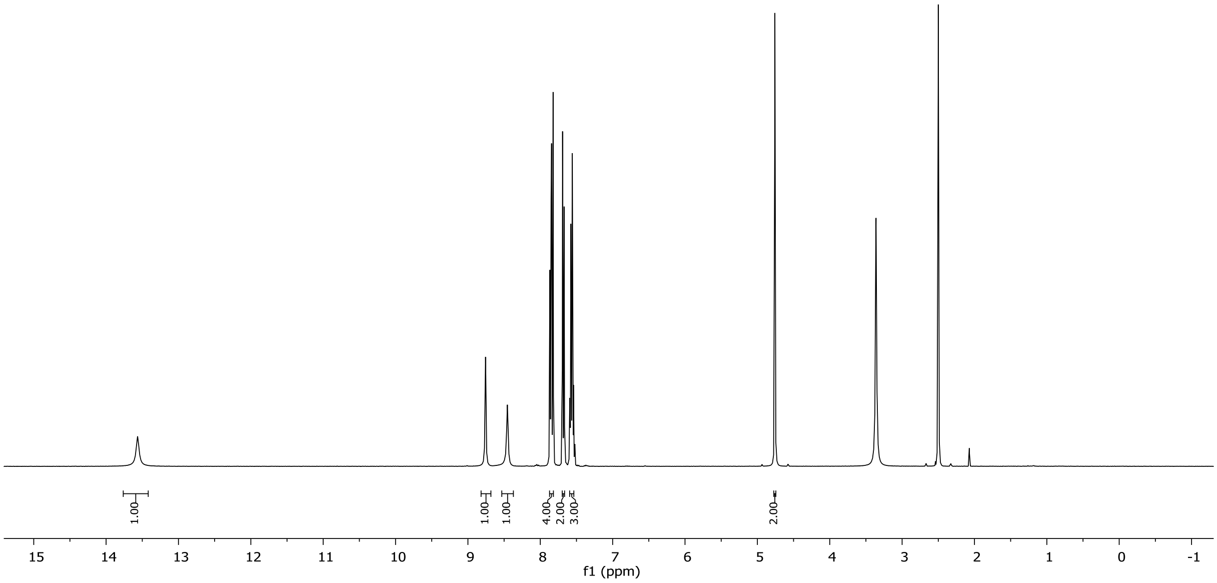

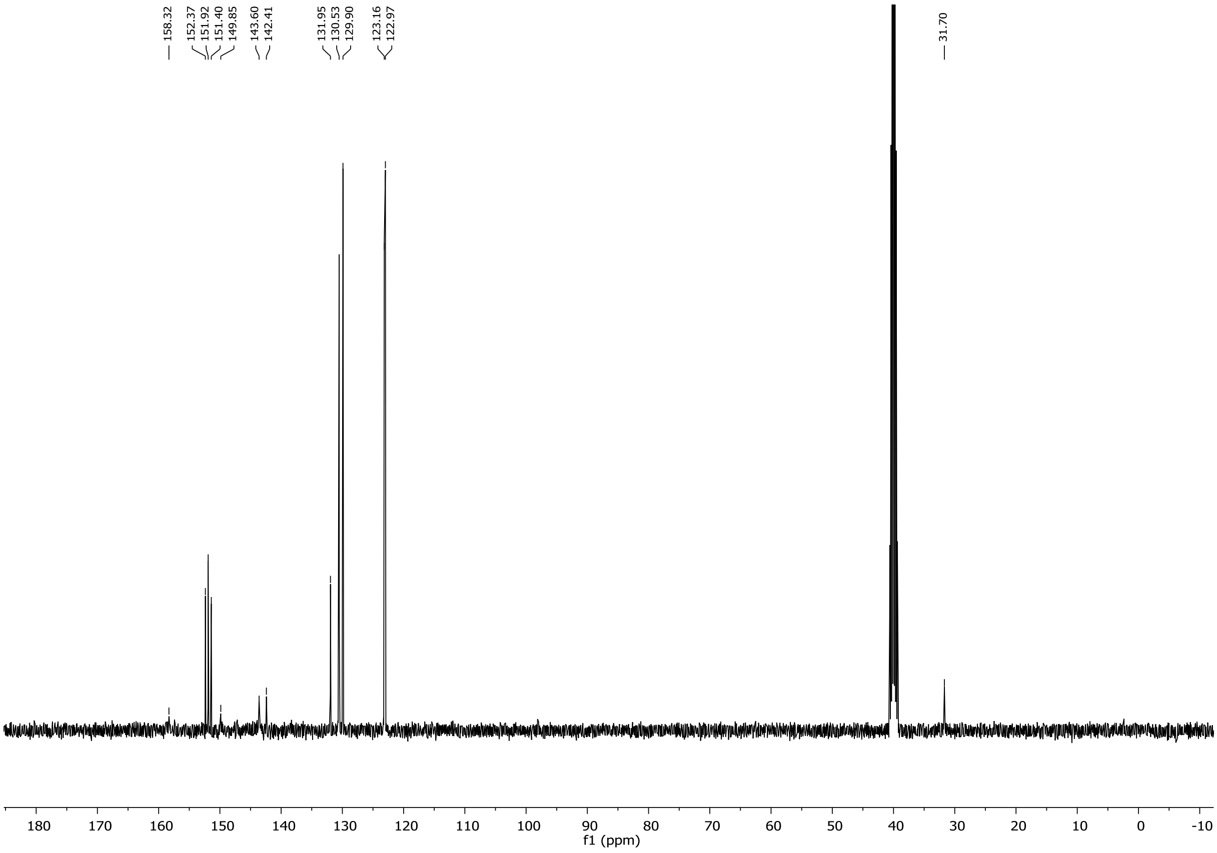

^{1}H and ^{13}C NMR spectra.

Compound **28** (DMSO-d_6)

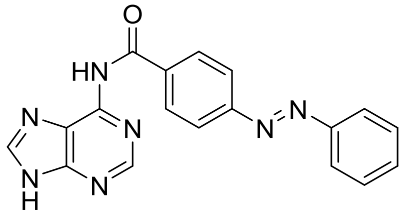

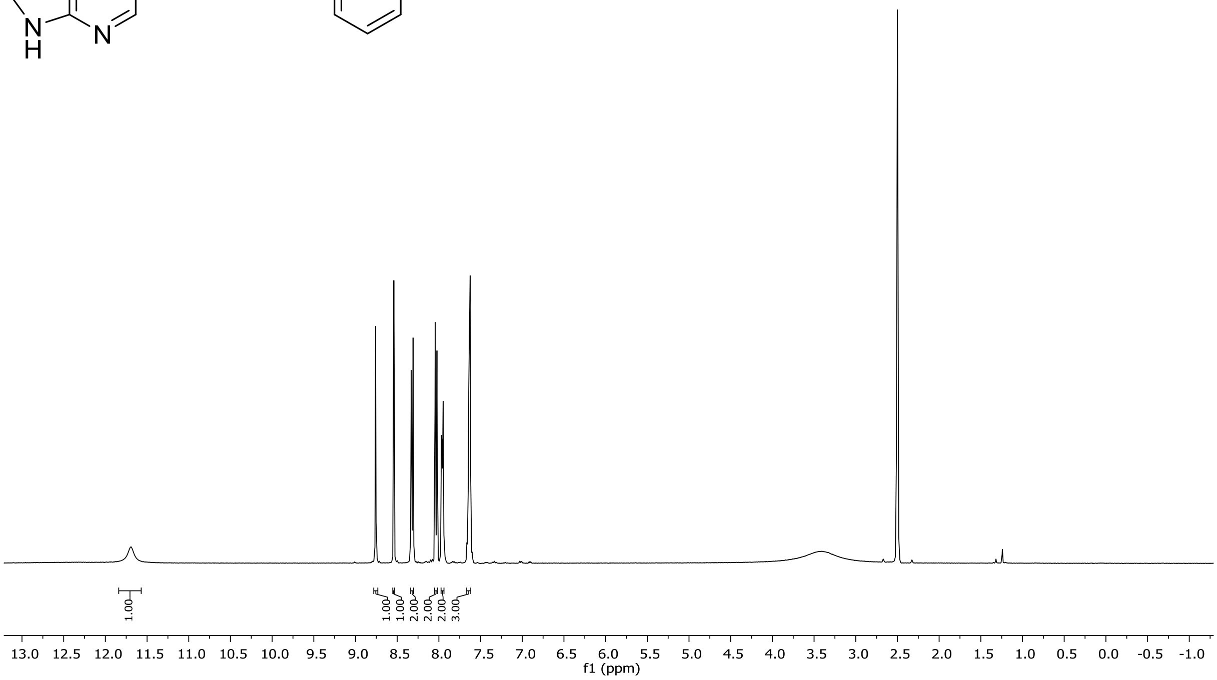

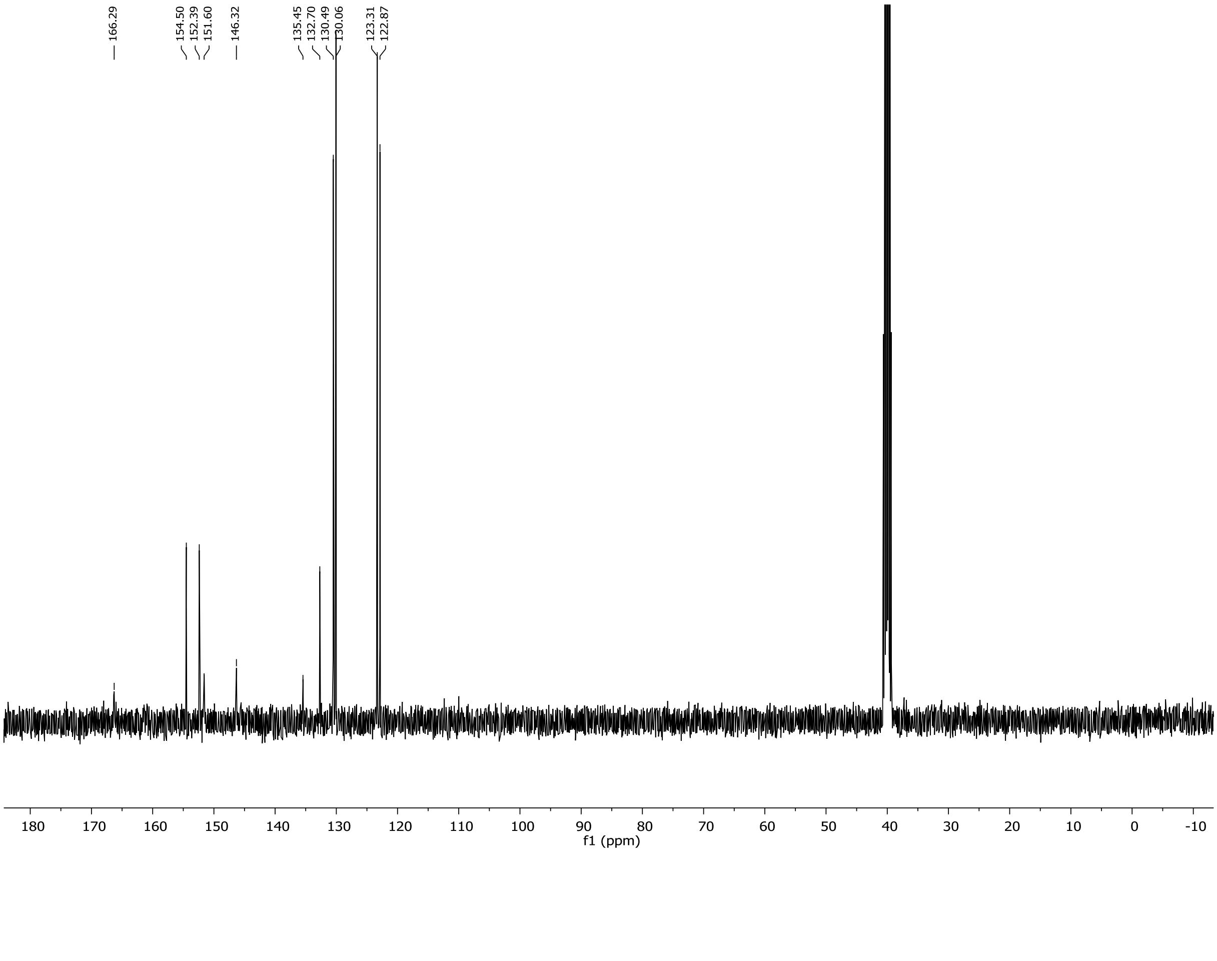

X-ray structures

Compound **12b**

Experimental. Single clear red plate-shaped crystals of **12b** were obtained by recrystallisation from acetone. A suitable crystal (0.088 × 0.195 × 0.309) mm^3 was selected and mounted on a MITIGEN holder with inert oil on a SuperNova, Single source at offset/far, Atlas diffractometer. The crystal was kept at T = 123 K during data collection. Using Olex2 (Dolomanov et al., 2009) [S1], the structure was solved with the ShelXT (Sheldrick, 2015) [S2] structure solution program, using the Intrinsic Phasing solution method. The model was refined with version 2016/6 of ShelXL (Sheldrick, 2015) [S3] using Least Squares minimisation.

Crystal Data. $C_{16}H_{14}N_4O$, M_r = 279.31, triclinic P-1 (No. 2), a = 7.3051(5) Å, b = 9.2030(6) Å, c = 11.3965(8) Å, α = 100.804(5)°, β = 97.060(5)°, γ = 113.159(6) °, V = 675.35(9) $Å^3$, T = 123(1) K, Z = 1 λ=(CuK_α) = 2.732, 395 reflections measured, 2567 unique (R_{int} = 0.0305) which were used in all calculations. The final wR_2 was 0.1266 (all data) and R_1 was 0.0522 (I > 2(I)).

Cambridge Structural Database CCDC: 1890055

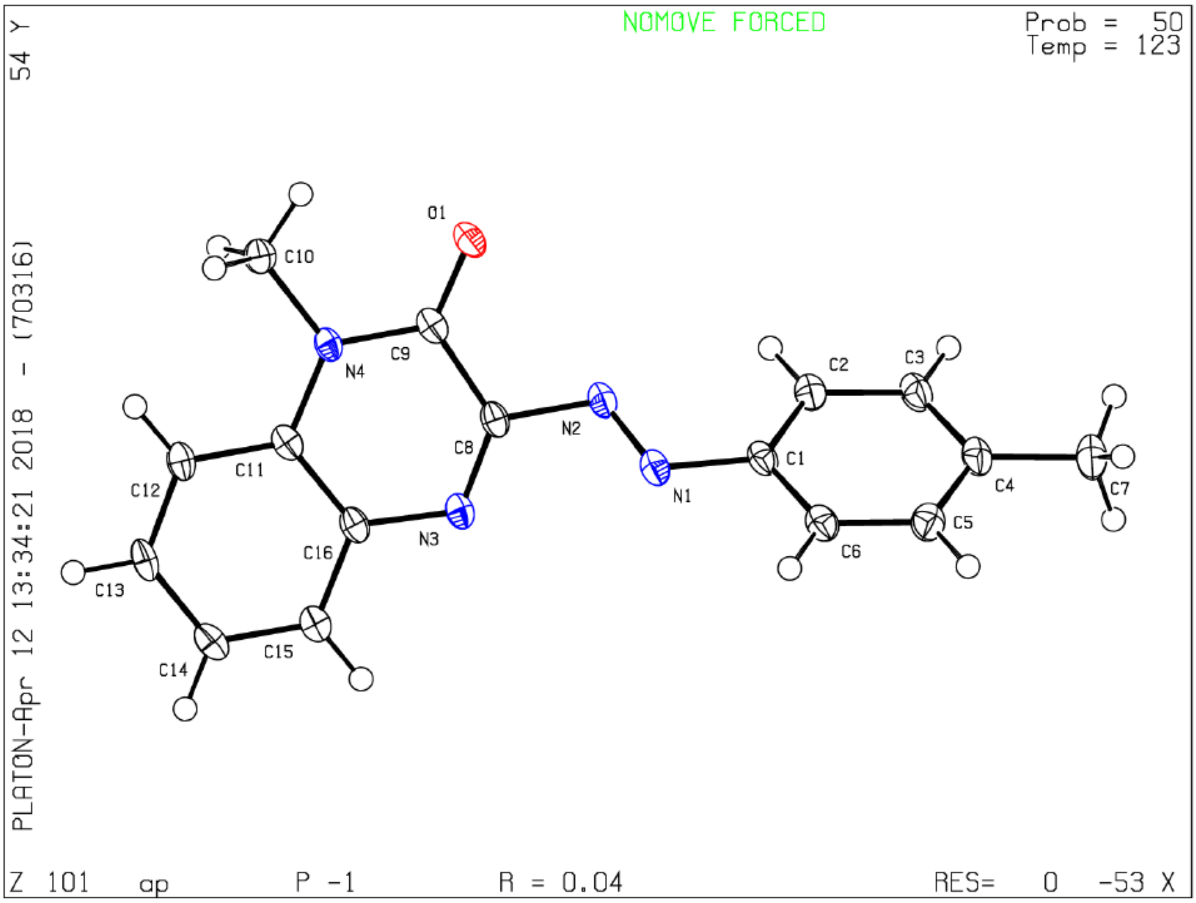

Detailed Crystal Data

Empirical formula	$C_{16}H_{14}N_4O$
Formula weight	278.31
Temperature/K	123(1)
Crystal system	triclinic
Space group	P-1
a/Å	7.3051(5)
b/Å	9.2030(6)
c/Å	11.3965(8)
α/°	100.804(5)
β/°	97.060(5)
γ/°	113.159(6)
Volume/Å^3	675.35(9)
Z	1
ρ_{calc}g/cm^3	0.684
μ/mm^{-1}	0.362
F(000)	146.0
Crystal size/mm^3	0.309 × 0.195 × 0.088
Radiation	CuKα (λ = 1.54184)
2Θ range for data collection/°	8.092 to 146.842
Index ranges	-8 ≤ h ≤ 8, -7 ≤ k ≤ 11, -14 ≤ l ≤ 11
Reflections collected	3951
Independent reflections	2567 [R_{int} = 0.0305, R_{sigma} = 0.0405]
Data/restraints/parameters	2567/0/192
Goodness-of-fit on F^2	1.036
Final R indexes [I>=2σ (I)]	R_1 = 0.0443, wR_2 = 0.1179
Final R indexes [all data]	R_1 = 0.0522, wR_2 = 0.1266
Largest diff. peak/hole / e Å^{-3}	0.24/-0.29

X-ray structure

Compound **16a**

Experimental. Single clear orange plate-shaped crystals of **16a** were obtained by recrystallisation from CH_2Cl_2. A suitable crystal 0.29 × 0.24 × 0.04 mm^3 was selected and mounted on a suitable support on an GV1000, TitanS2 diffractometer. The crystal was kept at a steady T = 123.01(13) K during data collection. The structure was solved with the **ShelXT** (Sheldrick, 2015)[S2] structure solution program using the Intrinsic Phasing solution method and by using **Olex2** (Dolomanov et al., 2009) [S1] as the graphical interface. The model was refined with version 2016/6 of **ShelXL** (Sheldrick, 2015) [S3] using Least Squares minimisation.

Crystal Data. $C_{12}H_8N_4S$, M_r = 240.28, monoclinic, $P2_1/n$ (No. 14), a = 7.0586(3) Å, b = 7.5744(2) Å, c = 20.8479(9) Å, β = 98.124(3)°, $\alpha = \gamma$ = 90°, V = 1103.44(7) Å^3, T = 123.01(13) K, Z = 4, Z' = 1, $\mu(CuK_\alpha)$ = 2.445, 7909 reflections measured, 2196 unique (R_{int} = 0.0606) which were used in all calculations. The final wR_2 was 0.1380 (all data) and R_1 was 0.0465 (I > 2(I)).

Cambridge Structural Database CCDC: 1889897

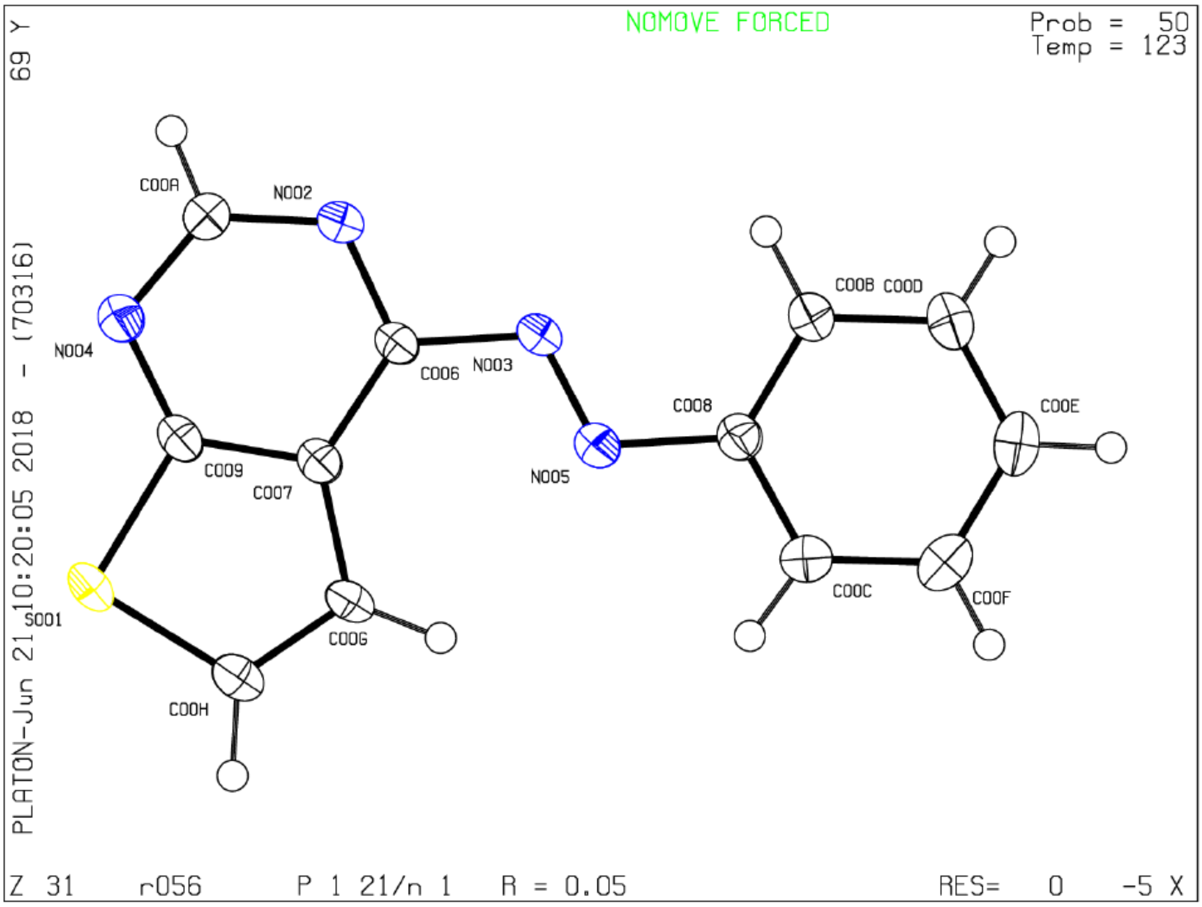

Detailed crystal data

Formula	$C_{12}H_8N_4S$
$D_{calc.}$/ g cm^{-3}	1.446
μ/mm^{-1}	2.445
Formula Weight	240.28
Colour	clear orange
Shape	plate
Size/mm^3	0.29×0.24×0.04
T/K	123.01(13)
Crystal System	monoclinic
Space Group	$P2_1/n$
a/Å	7.0586(3)
b/Å	7.5744(2)
c/Å	20.8479(9)
α/°	90
β/°	98.124(3)
γ/°	90
V/Å^3	1103.44(7)
Z	4
Z'	1
Wavelength/Å	1.54184
Radiation type	CuK[illegible]
Θ_{min}/°	4.284
Θ_{max}/°	74.411
Measured Refl.	7909
Independent Refl.	2196
Reflections with I > 2(I)	1981
R_{int}	0.0606
Parameters	186
Restraints	0
Largest Peak	0.344
Deepest Hole	-0.369
GooF	1.099
wR_2 (all data)	0.1380
wR_2	0.1321
R_1 (all data)	0.0516
R_1	0.0465

Experimental section

Chemistry

Commercially available reagents and starting materials were purchased from the commercial suppliers abcr, Acros Organics, Alfa-Aesar, Fisher Scientific, Merck, Sigma Aldrich, TCI, or VWR and used without any further purification. Solvents were used in p.a. quality and dried according to standard procedures, if necessary. Dry nitrogen was used as an inert gas atmosphere. Flash column chromatography was performed using Sigma Aldrich MN silica gel 60 M (40–63 µm, 230–400 mesh) for normal phase chromatography. Reaction monitoring via thin layer chromatography was performed on alumina plates coated with silica gel (Merck silica gel 60 F254, layer thickness 0.2 mm). Melting points were determined using a Stanford Research System OptiMelt MPA 100 and are uncorrected. NMR spectra were measured on a Bruker Avance 300 (^{1}H 300.13 MHz, ^{13}C 75.48 MHz), Bruker Avance III HD 400 (^{1}H 400.13 MHz, ^{13}C 100.61 MHz), Bruker Avance III HD 600 (^{1}H 600.25 MHz, ^{13}C 150.95 MHz) and Bruker Avance III 600 (^{1}H 600.25 MHz, ^{13}C 150.95 MHz). The spectra are referenced against residual non-deuterated NMR solvent signals (DMSO-d_6: δ_H = 2.50 ppm, δ_C = 39.52 ppm; $CDCl_3$-*d*: δ_H = 7.26 ppm, δ_C = 77.16 ppm) and chemical shifts δ are reported in ppm. Resonance multiplicity is abbreviated as: s (singlet), d (doublet), t (triplet) and m (multiplet). Carbon NMR signals are assigned using DEPT 135 and ^{1}H-^{13}C HSQC spectra with (+) for primary/tertiary, (−) for secondary, and (q) for quaternary carbons. Mass spectra were recorded on a Finnigan MAT-SSQ 710 A, ThermoQuest Finnigan TSQ 7000, Agilent Q-TOF 6540 UHD, or a Jeol AccuTOF GCX instrument. UV–vis absorption spectroscopy was performed in 10 mm quartz cuvettes using an Agilent 8543, Agilent Cary 100, or Agilent Varian Cary 50 spectrometer. Analytical HPLC measurements were performed using an Agilent 1220 Infinity LC (column: Phenomenex Luna 3 µm C18(2) 100 A, 150 x 2.00 mm; flow 0.3 mL min^{-1} at 20 °C or 30 °C; solvent A: MilliQ water with 0.05 wt % TFA; solvent B: MeCN). The ratios at the PSSs were determined via analytical HPLC at 20 °C at the isosbestic points or via NMR spectroscopy. An Agilent 1260 system (column: Phenomenex Luna 10 µm C18(2) 100 A, 250 x 21.2 mm; flow: 22 mL min^{-1}; solvent A: MilliQ water; solvent B: MeCN) was used for preparative HPLC purification. Light sources for irradiation: λ = 365 nm (Seoul Viosys CUN6GB1A, 1000 mA, 1.4 W), λ = 385 nm (Seoul Viosys CUN8GF1A, 1000 mA, 1.6 W), λ = 400 nm (Luxeon 400 nm SZ-01-S2, 500 mA, 0.48 W), λ = 455 nm (Osram OSLON SSL 80 LD-CQ7P-1U3U, 1000 mA, 0.45 W).The power of the light is given based on the specifications supplied by the company when the LEDs were purchased.

Methods

Chemistry

Compounds **2** [S4], **3** [S5], **6** [S6], **8** [S6], **10** [S7], **11** [S8], **14a**–**c** [S9], **19** [S10], **21** [S11], **25** [S12] and **26** [S13] were synthesized following adapted reported procedures.

(*E*)-2-(*p*-Tolyldiazenyl)quinoxaline (5a). This compound was synthesized via an adapted literature reported procedure [S5]. Nitrosoquinoxaline **3** (479 mg, 3.0 mmol, 1.0 equiv) and *p*-toluidine (**4a**, 362 mg, 3.4 mmol, 1.1 equiv) were suspended in acetic acid (10 mL), refluxed for 10 min and stirred at room temperature for additional 16 hours. Purification by flash column chromatography using CH_2Cl_2 as the eluent afforded the desired product as red solid (565 mg, 2.3 mmol, 76%). ^{1}H NMR (300 MHz, DMSO-d_6) δ = 9.32 (s, 1H), 8.26 – 8.19 (m, 2H), 8.01 – 7.96 (m, 4H), 7.50 (d, *J* = 8.6 Hz, 1H), 2.45 (s, 3H). ^{13}C NMR (75 MHz, DMSO) δ = 155.6 (q), 150.7 (q), 144.8 (q), 142.9 (q), 141.0 (q), 138.5 (+), 131.9 (+), 131.8 (+), 130.8 (+), 130.3 (+), 129.4 (+), 123.9 (+), 21.7 (+). m.p. 135 °C. HRMS (ESI) calcd. for ($C_{15}H_{13}N_4^+$) [M+H]$^+$: m/z = 249.1135; found 249.1138.

(*E*)-2-((3-Chloro-2-methylphenyl)diazenyl)quinoxaline (5b). This compound was synthesized via an adapted literature reported procedure [S5]. Nitrosoquinoxaline **3** (479 mg, 3.0 mmol, 1.0 equiv) and

aniline (**4b**, 479 mg, 3.4 mmol, 1.1 equiv) were mixed in acetic acid (10 mL) and heated to reflux for 10 min. The mixture was then heated at 50 °C for additional 16 hours. Purification by flash column chromatography using CH_2Cl_2 as the eluent afforded the target compound as red solid (40%). ^{1}H NMR (300 MHz, DMSO-d_6) δ = 9.35 (s, 1H), 8.32 – 8.18 (m, 2H), 8.06 – 7.94 (m, 2H), 7.74 (dd, *J* = 23.8, 8.1 Hz, 2H), 7.46 (t, *J* = 7.9 Hz, 1H), 2.81 (s, 3H). ^{13}C NMR (101 MHz, DMSO) δ = 155.6 (q), 151.7 (q), 143.1 (q), 141.0 (q), 138.4 (+), 137.9 (q), 135.7 (q), 133.9 (+), 132.2 (+), 131.9 (+), 130.3 (+), 129.5 (+), 128.3 (+), 114.5 (+), 14.7 (+). m.p. 140 °C. HRMS (ESI) calcd. for ($C_{15}H_{12}ClN_4^+$) $[M+H]^+$: m/z = 283.0745; found 283.0745.

(*E*)-2-Methoxy-3-(*p*-tolyldiazenyl)quinoxaline (12a) and **(*E*)-1-methyl-3-(*p*-tolyldiazenyl)quinoxalin-2(1*H*)-one (12b)**. These compounds were synthesized via an adapted literature reported procedure [S14]. Compound **11** (359 mg, 1.36 mmol, 1.0 equiv) and potassium carbonate (188 mg, 1.36 mmol, 1.0 equiv) were suspended in DMF (6 mL). Then methyl iodide (193 mg, 1.36 mmol, 1.0 equiv) was added and the mixture stirred at room temperature for 16 hours. Water (2 mL/mmol) was added to the mixture and the aqueous layer extracted with ethyl acetate for three times. The combined organic layers were washed with brine, dried over magnesium sulfate, filtered and the solvent evaporated. Purification by flash column chromatography using petroleum ether/ethyl acetate 1:1 as eluent afforded the products **12a** and **12b**. Characterization of **12a**. Red solid (45.4 mg, 0.163 mmol, 12%). Gradient 0-13 min: MeCN/H_2O 45:55 – 98:2, t_R = 12.2 min. ^{1}H NMR (600 MHz, DMSO-d_6) δ = 8.07 (dd, *J* = 8.2, 1.4 Hz, 1H), 7.97 – 7.88 (m, 3H), 7.82 (ddd, *J* = 8.4, 6.9, 1.5 Hz, 1H), 7.70 (ddd, *J* = 8.4, 7.0, 1.4 Hz, 1H), 7.48 (d, *J* = 8.1 Hz, 2H), 4.15 (s, 3H), 2.46 (s, 3H). ^{13}C NMR (75 MHz, $CDCl_3$) δ = 154.2 (q), 151.6 (q), 149.4 (q), 144.0 (q), 141.5 (q), 137.8 (q), 130.5 (+), 130.2 (+), 129.9 (+), 127.4 (+), 126.8 (+), 124.1 (+), 54.4 (+), 21.8 (+). m.p. 137 °C. HRMS (ESI) calcd. for ($C_{16}H_{15}N_4O^+$) $[M+H]^+$: m/z = 279.1240; found 279.1243. Characterization of **12b**. Orange solid (159 mg, 0.571 mmol, 42%). Gradient 0-10 min 45:55-90:10, t_R = 7.5 min. ^{1}H NMR (400 MHz, DMSO-d_6) δ = 7.87 (d, *J* = 8.4 Hz, 3H), 7.73 – 7.64 (m, 2H), 7.49 – 7.40 (m, 3H), 3.72 (s, 3H), 2.45 (s, 3H). ^{13}C NMR (101 MHz, DMSO) δ = 157.4 (q), 152.2 (q), 150.8 (q), 144.5 (q), 134.6 (q), 131.6 (+), 131.3 (q), 130.7 (+), 130.7 (+), 124.6 (+), 123.8 (+), 115.6 (+), 29.7 (+), 21.7 (+). m.p. 132 °C. HRMS (ESI) calcd. for ($C_{16}H_{15}N_4O^+$) $[M+H]^+$: m/z = 279.1240; found 279.1243. Cambridge Structural Database CCDC: 1890055

(*E*)-4-(Phenyldiazenyl)thieno[2,3-*d*]pyrimidine (16a). This compound was synthesized via an adapted literature reported procedure [S15]. 4-Chlorothieno[2,3-*d*]pyrimidine (**15c**, 200 mg, 1.2 mmol, 1.0 equiv), phenylhydrazine (**13b**, 152 mg, 1.4 mmol, 1.2 equiv), DIPEA (758 mg, 5.9 mmol, 5.0 equiv) and *n*-BuOH (5 mL) were mixed and stirred at 150 °C for 16 hours. The reaction mixture was cooled to room temperature and exposed to pure oxygen (balloon) for 24 hours. Purification by flash column chromatography using CH_2Cl_2 as the eluent afforded the desired product as red solid (151 mg, 0.6 mmol, 53%). ^{1}H NMR (400 MHz, DMSO-d_6) δ = 9.25 (s, 1H), 8.18 (d, *J* = 6.0 Hz, 1H), 8.13 – 8.10 (m, 2H), 7.89 (d, *J* = 6.0 Hz, 1H), 7.74 – 7.69 (m, 3H). ^{13}C NMR (101 MHz, DMSO) δ = 172.1 (q), 161.4 (q), 153.7 (+), 152.7 (q), 134.4 (+), 131.9 (+), 130.3 (+), 124.0 (+), 121.7 (q), 120.6 (+). m.p. 106 °C. HRMS (ESI) calcd. for ($C_{12}H_9N_4S^+$) $[M+H]^+$: m/z = 241.0542; found 241.0540. Cambridge Structural Database CCDC: 1889897

(*E*)-6-((2-Chlorophenyl)diazenyl)-9*H*-purine (16b). This compound was synthesized via an adapted literature reported procedure [S15]. 6-Chloro-9-isopropyl-9*H*-purine (371 mg, 2.4 mmol, 1.0 equiv), *o*-chlorophenylhydrazine (411 mg, 2.9 mmol, 1.2 equiv), DIPEA (1.55 g, 12 mmol, 5.0 equiv) and *n*-BuOH (16 mL) were mixed and stirred in a glass vial at 150 °C for 16 hours. The reaction mixture was cooled to room temperature and exposed to pure oxygen (balloon) for 24 hours. The solvent was removed and the product purified by flash column chromatography using CH_2Cl_2 + 5% MeOH to afford **16b** as red solid (417 mg, 1.6 mmol, 67%). ^{1}H NMR (400 MHz, DMSO-d_6) δ = 13.26 (s, 1H), 9.10 (s, 1H), 8.83 (s, 1H), 7.82 – 7.78 (m, 2H), 7.73 – 7.69 (m, 1H), 7.60 – 7.55 (m, 1H). ^{13}C NMR (101 MHz, DMSO) δ = 152.3 (+), 149.0 (q), 135.8 (q), 135.2 (+), 131.5 (+), 128.7 (+), 118.2 (+). m.p. 179 °C. HRMS (ESI) calcd. for ($C_{11}H_8ClN_6^+$) $[M+H]^+$: m/z = 259.0493; found 259.0493.

(*E*)-6-(Naphthalen-1-yldiazenyl)-9*H*-purine (16c). This compound was synthesized via an adapted literature reported procedure [S15]. A mixture of chloroadenine **15a** (93.0 mg, 0.60 mmol, 1.0 equiv), hydrazine **14b** (114 mg, 0.72 mmol, 1.2 equiv) and DIPEA (388 mg, 0.52 mL, 3.0 mmol, 5.0 equiv) in *n*-butanol (4.0 mL) was stirred at 150 °C for 16 hours. After cooling to room temperature, the solution was exposed to an oxygen atmosphere (balloon) for 24 hours. The product was purified by column chromatography using CH_2Cl_2 + 5% MeOH as eluent and subsequent preparative HPLC (gradient 0-20 min: MeCN/H_2O 10:90 – 98:2) and afforded adenine-azo **16c** as red solid (t_R = 11.47 min, 92.0 mg, 0.37 mmol, 62%). NMR (400 MHz, DMSO-d_6) δ = 13.53 (s, 1H), 9.14 (s, 1H), 8.91 (d, *J* = 8.4 Hz, 1H), 8.85 (s, 1H), 8.31 (d, *J* = 8.0 Hz, 1H), 8.13 (d, *J* = 8.0 Hz, 1H), 7.99 (d, *J* = 7.5 Hz, 1H), 7.80 – 7.70 (m, 3H). ^{13}C NMR (151 MHz, DMSO) δ = 155.0 (q), 151.8 (+), 149.3 (q), 147.7 (+), 134.0 (+), 133.9 (q), 130.2 (q), 128.2 (+), 128.0 (+), 127.0 (+), 125.8 (+), 123.3 (+), 113.6 (q). m.p. 110 °C. HRMS (ESI) calcd. for ($C_{15}H_{11}N_6^+$) $[M+H]^+$: m/z = 275.1040; found 275.1044.

(*E*)-9-Methyl-6-(naphthalen-1-yldiazenyl)-9*H*-purine (16d). This compound was synthesized via an adapted literature reported procedure [S15]. A mixture of methylated chloroadenin **15b** (101 mg, 0.60 mmol, 1.0 equiv), hydrazine **14b** (114 mg, 0.72 mmol, 1.2 equiv) and DIPEA (388 mg, 0.52 mL, 3.0 mmol, 5.0 equiv) in *n*-butanol (4.0 mL) was stirred at 150 °C for 16 hours. After cooling to room temperature, the solution was exposed to an oxygen atmosphere (balloon) for 24 hours. The product was purified by column chromatography using CH_2Cl_2 + 5% MeOH as eluent and subsequent preparative HPLC (gradient 0-20 min: MeCN/H_2O 10:90 to 98:2) and afforded the methylated azoadenine **16d** as red solid (t_R = 12.91 min, 130 mg, 0.45 mmol, 75%). ^{1}H NMR (400 MHz, Chloroform-*d*) δ = 9.13 (s, 1H), 9.08 (d, *J* = 7.3 Hz, 1H), 8.27 (s, 1H), 8.09 (d, *J* = 7.8 Hz, 2H), 7.94 (d, *J* = 7.8 Hz, 1H), 7.71 – 7.66 (m, 1H), 7.62 – 7.58 (m, 2H), 4.00 (s, 3H). ^{13}C NMR (101 MHz, $CDCl_3$) δ = 158.0 (q), 155.6 (q), 152.7 (+), 148.6 (q), 147.5 (+), 134.3 (q), 134.1 (+), 132.0 (q), 127.9 (+), 127.9 (+), 126.8 (+), 126.0 (q), 125.4 (+), 123.9 (+), 113.0 (+), 30.2 (+). m.p. 186 °C. HRMS (ESI) calcd. for ($C_{16}H_{13}N_6^+$) $[M+H]^+$: m/z = 289.1196; found 289.1198.

(*E*)-6-((4-(Phenyldiazenyl)benzyl)thio)-7*H*-purine (23). This compound was synthesized via an adapted literature reported procedure [S16]. A solution of chloromethylated azobenzene **21** (800 mg, 3.5 mmol, 1.1 equiv) in DMF (10 mL) was added to a solution of 6-mercaptopurine **22** (151 mg, 3.2 mmol, 1.0 equiv) in 2 M NaOH (10 mL) and the mixture stirred at room temperature for 3 hours. The solvent was evaporated and the product purified by flash column chromatography using CH_2Cl_2 + 5% MeOH as the eluent. Evaporation of the solvent afforded the desired product as orange solid (215 mg, 0.62 mmol, 18%). ^{1}H NMR (400 MHz, DMSO-d_6) δ = 13.55 (s, 1H), 8.76 (s, 1H), 8.46 (s, 1H), 7.89 – 7.83 (m, 4H), 7.68 (d, *J* = 8.4 Hz, 2H), 7.59 – 7.55 (m, 3H), 4.76 (s, 2H). ^{13}C NMR (101 MHz, DMSO) δ = 158.3 (q), 152.4(q), 151.9 (+), 151.4 (q), 149.9 (q), 143.6 (q), 142.4 (q), 132.0 (+), 130.5 (+), 129.9 (+), 123.2 (+), 123.0 (+), 31.7 (-). m.p. 212 °C. HRMS (ESI) calcd. for ($C_{18}H_{15}N_6S^+$) $[M+H]^+$: m/z = 347.1073; found 347.1077.

(*E*)-4-(Phenyldiazenyl)-*N*-(9*H*-purin-6-yl)benzamide (28). This compound was synthesized via an adapted literature reported procedure [S17]. Chlorocarbonylazobenzene **26** (636 mg, 2.6 mmol, 1.1 equiv) was added dropwise over 30 min to a stirred suspension of adenine **27** (324 mg, 2.4 mmol, 1.0 equiv) in dry pyridine and stirring was continued for two hours at 100 °C. The mixture was cooled to room temperature and stirred for additional 16 hours. The reaction was quenched with methanol and the solvents were removed under reduced pressure. Purification by column chromatography using CH_2Cl_2 as the eluent afforded the desired product as orange solid (684 mg, 2.0 mmol, 83%). ^{1}H NMR (400 MHz, DMSO-d_6) δ = 11.69 (s, 1H), 8.76 (s, 1H), 8.54 (s, 1H), 8.32 (d, *J* = 8.3 Hz, 2H), 8.03 (d, *J* = 8.3 Hz, 2H), 7.97 – 7.94 (m, 2H), 7.67 – 7.62 (m, 3H). ^{13}C NMR (101 MHz, DMSO) δ = 166.2 (q), 154.5 (q), 152.4 (q), 151.6 (+), 146.3 (+), 135.5 (q), 132.7 (+), 130.5 (+), 130.1 (+), 123.3 (+), 122.9 (+). m.p. 271 °C. HRMS (ESI) calcd. for ($C_{18}H_{14}N_7O^+$) $[M+H]^+$: m/z = 344.1254; found 344.1257.

Cell culture and transfection

The subtype A of 5-HT_3receptors was heterologously expressed in cultured Chinese hamster ovary (CHO) cells obtained from the American Type Tissue Culture Collection (ATCC, Molsheim, France). Transfection with cDNA of 5-HT_3A receptors was performed using the Lipofectamine 3000 protocol (Life Technology, USA). For identification of transfected cells a cDNA of green fluorescent protein (GFP) was co-transfected with cDNA of 5-HT_3ARs. Three hours after the initial exposure of cells to the cDNAs the culture medium was replaced with fresh medium. Electrophysiological recordings were carried out on fluorescent cells 24–72 hours after transfection.

Electrophysiological recordings

Whole-cell patch-clamp recordings were held at room temperature (20–25 ^oC) using an EPC-9 amplifier (HEKA Elektronik, Germany). Cells were continuously superfused with external solution containing (mM): NaCl 140, $CaCl_2$ 2, KCl 2.8, $MgCl_2$ 4, HEPES 20, glucose 10; pH 7.4; 320–330 mOsm. Intracellular solution used for filling recording patch pipettes contained (mM): KCl 140, $MgCl_2$ 2, MgATP 2, BAPTA (tetrapotassium salt) 2; pH 7.3; 290 mOsm. Recording pipettes were pulled from borosilicate glass capillaries (Harvard Apparatus Ltd, USA) and had resistances of 5–10 MOhm. Rapid replacement of solutions was provided by fast application system (SF 77A Perfusion Fast-Step, Warner, USA), placed 40–50 µm above the recorded cell. Cells with low input resistance (<150 MOhm) and a rapid run-down (>30% with repetitive application) were excluded from analysis. The agonist 5-HT for the activation was applied alone or mixed with studied compounds during 5 s. Irradiation with the light of 455 nm or 530 nm was provided by LEDs (Thorlabs) placed at the distance of 4–5 cm from the studied cells.

References

[S1] O.V. Dolomanov and L.J. Bourhis and R.J. Gildea and J.A.K. Howard and H. Puschmann, Olex2: A complete structure solution, refinement and analysis program, *J. Appl. Cryst.* **2009**, *42*, 339-341.

[S2] Sheldrick, G.M., ShelXT-Integrated space-group and crystal-structure determination, *Acta Cryst.*, **2015**, *A71*, 3-8.

[S3] Sheldrick, G.M., Crystal structure refinement with ShelXL, *Acta Cryst.* **2015**, *C27*, 3-8.

[S4] M. von Wantoch Rekowski, A. Pyriochou, N. Papapetropoulos, A. Stößel, A. Papapetropoulos, A. Giannis, *Bioorg. Med. Chem.* **2010**, *18*, 1288-1296.

[S5] K. Harsányi, C. Gönczi, D. Korbonits, *Liebigs Ann. Chem.* **1973**, *2*, 190-194.

[S6] S. Murarka, P. Martín-Gago, C. Schultz-Fademrecht, A. Al Saabi, M. Baumann, E. K. Fansa, S. Ismail, P. Nussbaumer, A. Wittinghofer, H. Waldmann, *Chem. Eur. J.* **2017**, *23*, 6083-6093.

[S7] V. Colotta, D. Catarzi, F. Varano, L. Cecchi, G. Filacchioni, A. Galli, C. Costagli, *Arch. Pharm. Pharm. Med. Chem.* **1997**, *330*, 387-391.

[S8] C. Párkányi, A. O. Abdelhamid, *J. Heterocyclic Chem.* **1984**, *21*, 521-524.

[S9] X. Wang, Y.-F. Chen, W. Yan, L.-L. Cao, Y.-H. Ye, *Molecules* **2016**, *21*, 1574-1588.

[S10] P. Stawski, M. Sumser, D. Trauner, *Angew. Chem. Int. Ed.* **2012**, *51*, 5748-5751.

[S11] J. del Barrio, P. N. Horton, D. Lairez, G. O. Lloyd, C. Toprakcioglu, O. A. Scherman, *J. Am. Chem. Soc.* **2013**, *135*, 11760-11763.

[S12] F.-N. Meng, Z.-Y. Li, Y.-L. Ying, S.-C. Liu, J. Zhang, Y.-T. Long, *Chem. Com.* **2017**, *53*, 9462-9465.

[S13] G. H. Coleman, G. Nichols, C. M. McCloskey, H. D. Anspon, *Organic Syntheses* **1945**, *25*, 87-89.

[S14] S. Pierau, G. Dale, *Assignee Morphochem Aktiengesellschaft fuer Kombinatorische Chemie Germany* **2006**, *WO 2006021448 A1.*

[S15] D. Kolarski, W. Szymanski, B. L. Feringa, *Org. Lett.* **2017**, *19*, 5090-5093.

[S16] S. A. Laufer, D. M. Domeyer, T. R. F. Scior, W. Albrecht, D. R. J. Hauser, *J. Med. Chem.* **2005**, *48*, 710-722.

[S17] C. F. Liu, Y. Zeng, X. W. Lu, *Assignee Nanyang, Technological University* **2010**, *WO2010027326 A1.*
